# Chirality Matters: Biological Activity of Optically Pure Silybin and Its Congeners

**DOI:** 10.3390/ijms22157885

**Published:** 2021-07-23

**Authors:** Vladimír Křen

**Affiliations:** Laboratory of Biotransformation, Institute of Microbiology of the Czech Academy of Sciences, Vídeňská 1083, 142 20 Prague, Czech Republic; kren@biomed.cas.cz; Tel.: +420-296-442-510

**Keywords:** silymarin, silybin, *Silybum marianum*, milk thistle, diastereomer, chirality, isosilybin, silychristin, silydianin, silibinin, flavonolignan, flavonoid, dehydroflavonolignan

## Abstract

This review focuses on the specific biological effects of optically pure silymarin flavo-nolignans, mainly silybins A and B, isosilybins A and B, silychristins A and B, and their 2,3-dehydro derivatives. The chirality of these flavonolignans is also discussed in terms of their analysis, preparative separation and chemical reactions. We demonstrated the specific activities of the respective diastereomers of flavonolignans and also the enantiomers of their 2,3-dehydro derivatives in the 3D anisotropic systems typically represented by biological systems. In vivo, silymarin flavonolignans do not act as redox antioxidants, but they play a role as specific ligands of biological targets, according to the “lock-and-key” concept. Estrogenic, antidiabetic, anticancer, antiviral, and antiparasitic effects have been demonstrated in optically pure flavonolignans. Potential application of pure flavonolignans has also been shown in cardiovascular and neurological diseases. Inhibition of drug-metabolizing enzymes and modulation of multidrug resistance activity by these compounds are discussed in detail. The future of “silymarin applications” lies in the use of optically pure components that can be applied directly or used as valuable lead structures, and in the exploration of their true molecular effects.

## 1. Introduction

Flavonoids are generally considered to have potent antioxidant and antiradical activities. This is true under in vitro conditions when these compounds can reach concentrations over 10–100 µM. However, in vivo they can hardly reach plasma concentrations over 1–5 µM, due to their low water solubility and bioavailability. The often measured antioxidant capacity of phytochemicals is actually an index of their sensitivity to oxidation rather than an index of the protection they can elicit in vivo. It is well documented in the modern literature and in numerous clinical studies that plant “antioxidants” cannot achieve the postulated effects [1]. However, nobody can deny that flavonoids possess numerous valuable biological activities—observed in vivo. This paradox is well explained by the concept of para-hormesis [2], which hypothesizes that “antioxidants” act in a prooxidative manner to activate nuclear factor Nrf2, which then triggers the synthesis of “true antioxidants” in the cell, e.g., enzymatic cytoprotective systems.

Therefore, it is obvious that plant antioxidants including polyphenols do not act in vivo as mere chemical antioxidants active in an isotropic milieu, but in fact, they act as effectors on the cellular receptors. For a proper interaction with the receptor, the respective complementary 3D structure of the ligand is required, which implies that the correct stereochemistry of the respective “antioxidant” plays a pivotal role.

**Figure 1:**
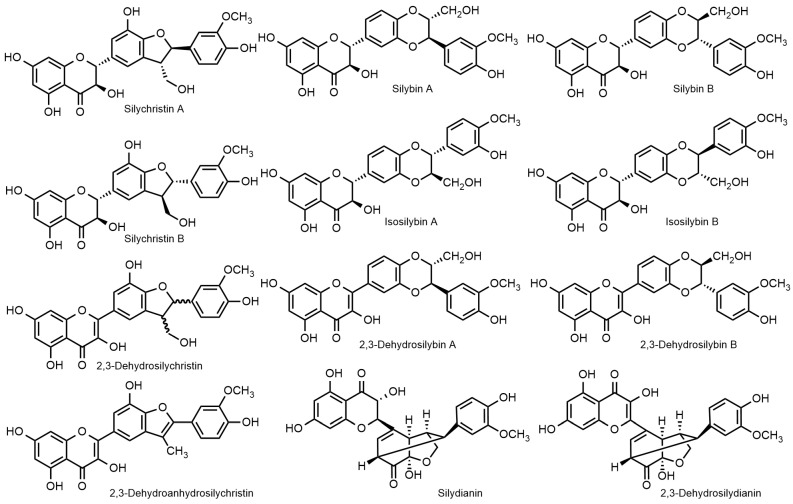
Major flavonolignans of silymarin.

Flavonolignans from the milk thistle (*Silybum marianum*), including silybin and 2,3-dehydrosilybin, are characterized by the occurrence of two to four chiral centers (Figure 1). They exist in nature as various stereomers, and in numerous recent studies it has been demonstrated that stereochemistry matters when describing their biological effects. Moreover, despite a plethora of reports describing these compounds as “potent antioxidants”, e.g., [3], in reality, they are truly poor antioxidants [4]. Additionally, many studies completely ignore the existence of stereomers, which leads to misleading or entirely wrong results.

The purpose of this review is to demonstrate the importance of working with optically pure stereomers of flavonolignans. We will show radical differences in the respective chemical and biological activities of stereomers of silybin and 2,3-dehydrosilybin and their pure congeners.

## 2. Silymarin and Silymarin Flavonolignans

Silymarin is a crude extract from fruits (cypselae, achenes; often incorrectly denoted as seeds) of the milk thistle *Silybum marianum* (L.) Gaertn., Asteraceae var. purple [5]. Silymarin is obtained from the fruits, which are pressed to remove most of the fats and further defatted with petroleum ether. The resulting cake is typically extracted with acetone or ethyl acetate, and the remaining fat is separated after dilution with water. Silybin is then obtained from the silymarin solution by precipitation with ethanol [6]. In addition to *S. marianum* var. *purple*, there is also *S. marianum* var. *albiflora* (white flowering), which produces different types of flavonolignans, such as, e.g., silandrin, isosilandrin, silymonin, and silyamandin [7].

Silymarin, being a crude extract of plant material, has a high degree of variability. It typically contains 20–30% polymeric phenolic fraction, ca. 30% silybin (as a quasi-equimolar mixture of silybin A and B), and other flavonolignans: ca. 5% isosilybin A and B, ca. 7% silychristin A, and ca. 10% silydianin. In addition to flavonolignans, silymarin contains 2–5% taxifolin, ca. 3% 2,3-dehydroflavonolignans, and some other polyphenolic compounds (Figure 1). The composition of silymarin depends on many factors such as the plant origin (chemorace—cf. [8]) cultivation and processing. We reviewed the issues of silymarin composition [9]. The recently developed advanced analytical methods [10,11] now enable the analysis of virtually all major silymarin components, including dehydroflavonolignans, in a single run; moreover, these methods enable a baseline-separation of respective diastereomers. A high variability of silymarin composition was demonstrated in a panel of various commercial preparations using this method [10].

The composition of silymarin and also the respective nomenclature of its components is a source of many controversies [12]. It is imperative to work with well-defined silymarin preparations or, much better, with (optically) pure flavonolignan components [13,14,15]. Not adhering to these principles generates a lot of unreproducible results in the literature, or even scientifically wrong concepts. The use of mixed silybin A and B may be also one explanation for the often observed pleiotropic effect of this substance. Regrettably, the literature is full of errors; even a basic reference book—Merck Index 14th Ed. [16]—presents the structure of silybin (silibinin) as a single compound (silibinin A), completely ignoring the existence of two stereomers. This mistake was corrected in the Merck Index 15th Ed. [17], where both silybin A and B were already presented correctly.

### 2.1. Nomenclature of Silybin Diastereomers

The nomenclature of silymarin flavonolignans is a rather intriguing problem and has been reviewed/revised by the authors who are well aware of the full complexity of these molecules. Unfortunately, typically biologists and physicians often ignore standard nomenclature and use various names for different preparations, which is often a cause of controversies in the literature. We pointed out this problem already, in 2000, in the Hepatology editorial [13]. Formally, these compounds are not lignans or neolignans, because only one intact phenylpropanoid unit is involved in the radical coupling. However, also due to biogenetic origin, alternative terms such as flavonolignoids [18] and lignoflavonoids [19] are also correct.

Silybin (silybum substance E6) was described for the first time and named in the pioneering paper by Pelter and Hansel [20]. Its synonym silibinin later appeared in pharmacopeias and, hence, it became preferential in the medical and pharmacological literature. In the paper on silybin nomenclature [12], the authors arbitrarily coined this name for a description of natural silybin (mixture of stereomers), while completely ignoring the etymology of the word silibinin and the fact that two names were originally used (and still being used) as synonyms. The synonym silibinin was thus given a different meaning, which is rather puzzling for the common users of this substance. Extending this concept to isosilybin (isosilybinin) might be feasible, but for silychristin (silichristinin?) it would sound weird. There is an additional aspect to this problem: natural silybin is not an equimolar mixture of silybin A and B, but silybin B predominates with a proportion of up to 7:3 [10]. This ratio depends on the silymarin source and processing [9], and it can be even changed by crystallization. Anyway, the literature on natural silybins should always provide their exact composition.

**Figure 2:**
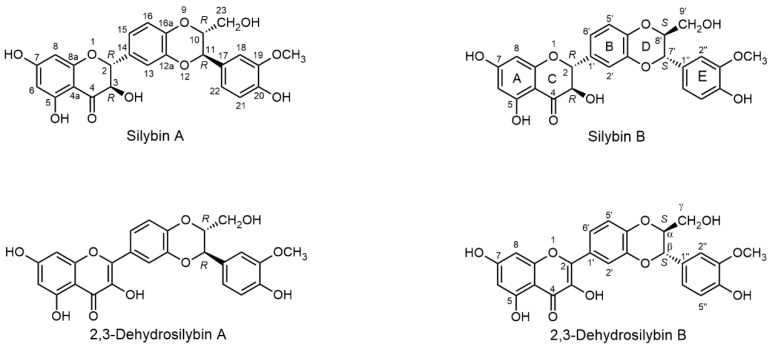
Structures and numbering styles of stereomers of silybin and 2,3-dehydrosilybin: The structure of silybin A is numbered according to a proprietary numbering system used in most chemical papers and also used in this review; the structure of silybin B is numbered according to IUPAC systematic numbering, and for the structure of 2,3-dehydrosilybin B, there is a quasi-systematic numbering used by some groups in the USA [12,21,22]; in some papers, the α-β numbering can be swapped. Other numbering systems used in the older literature for silybin only are detailed in the Supplementary Materials of the paper by Napolitano et al. [23].

The Merck Index and Chemical Abstracts are standards of chemical and pharmacological nomenclature: Merck Index 14th Ed. [16] only uses the name silybin, whereas Edition 15 [17] admitted the existence of silybin A and B and isosilybin A and B (entry #8532) and gives the name silibinin as the only one of the many synonyms of silybin (e.g., silibin; silibinin; silybum substance E6; silymarin I). Chemical Abstracts, under entry CAS No. 22888-70-6, give the priority name “silybin” (plus further synonyms). Therefore, it is appropriate to use standard names recommended in this literature (Merck Index, Chem. Abstr.), i.e., silybin (diastereomeric mixture), silybin A ((2*R*,3*R*)-2-((2*R*,3*R*)-2,3-dihydro-3-(4-hydroxy-3-methoxyphenyl)-2-(hydroxymethyl)-1,4-benzodioxin-6-yl)-2,3-dihydro-3,5,7-trihydroxy-4*H*-1-benzopyran-4-one) and silybin B [(2*R*,3*R*)-2-((2*S*,3*S*)-2,3-dihydro-3-(4-hydroxy-3-methoxyphenyl)-2-(hydroxy-methyl)-1,4-benzodioxin-6-yl)-2,3-dihydro-3,5,7-trihydroxy-4*H*-1-benzopyran-4-one] for the respective diastereomers (Figure 2).

Silybin A and B can be easily distinguished by HPLC and co-chromatography with authentic standards, e.g., using the modern HPLC method by Petrásková et al. [10]. Optical rotation can clearly discriminate between silybin A and B [6], since silybin A (${\left[\alpha \right]}_{589}^{20}$ = +20.0 (*c* 0.3, acetone), [24]) has a considerably higher optical rotation than silybin B (${\left[\alpha \right]}_{589}^{20}$ = −1.07 (*c* 0.3, acetone), [24]). Nevertheless impurities such as *cis*-silybins [25] (e.g., (2*R*,3*S*,10*R,*11*R*)*-*silybin ${\left[\alpha \right]}_{589}^{20}$ = −51.6 (*c* 0.091, acetone)), which have optical rotation considerably different from natural silybins, can strongly influence the results. NMR measurements are not quite suitable, as both diastereomers have very similar spectra; even though two sets of similar signals are observable in the ^13^C high-resolution NMR spectra of natural silybin, it is impossible to assign the respective diastereomers (apart from using authentic standards).

### 2.2. Biosynthesis of Silymarin Flavonolignans

From the early stages of the investigation into silymarin chemistry in the 1950s, it was assumed that flavonolignans of silymarin are formed by nonregio-, and nonstereoselective coupling between taxifolin and coniferyl alcohol. Catalysis by an unknown peroxidase was proposed, as well as a radical mechanism of the reactions. This was corroborated in 1977 in the early work by Schrall and Becker [26]. The authors prepared silybin by feeding taxifolin and coniferyl alcohol to a suspension culture of *S. marianum*. The action of the horseradish peroxidase on the mixture of coniferyl alcohol and taxifolin also yielded silybin. These observations enabled the authors to present the first tentative mechanism of silybin formation. However, due to an insufficient knowledge of silymarin flavonolignans and the limited analytic capabilities at that time (only IR and MS were available), the results were not interpreted correctly, nor were the conclusions confirmed by other studies. Instead of pure silybin, these authors obtained a mixture of silybin and isosilybin diastereomers (identical mass, similar IR spectra) as confirmed by recent reports [27]. Later, Merlini et al. [28] tested a biomimetic reaction using Ag_2_O as a radical initiator. It became clear, as postulated by Schrall and Becker [26] and unambiguously confirmed by Merlini et al. [28], that the enzymatic catalysis is only required for the generation of the radicals and not for their coupling, which is spontaneous. The radicals of both taxifolin and coniferyl alcohol are required for the generation of silybin/isosilybin diastereomers [29]. Two groups of enzymes—laccases and peroxidases—were assumed to produce the radicals for flavonolignan synthesis [30]. However, laccase was later excluded [31], and it was demonstrated that silybin is synthesized from taxifolin and coniferyl alcohol, with the terminal step catalyzed by ascorbate peroxidase (APX1). Taxifolin and coniferyl alcohol are bio-synthesized via the classical flavonoid and monolignol pathways, respectively, and the analysis of gene expression and metabolite levels at different stages of *S. marianum* fruit ripening revealed that the formation of flavonoid precursors precedes flavonolignan biosynthesis [32]. It was also established that the maximum activity of laccase is in the plant leaves and not in the blooming part that produces fruits [31]. Recently, Lv et al. [27] established that the ascorbate peroxidase responsible for the biosynthesis of silymarin flavonolignans is not stereoselective. This specific finding and general non-stereoselective feature of radical reactions clearly resonates with the situation that flavonolignans occur as pairs of diastereomers. The radical nature of the biosynthesis is also responsible for the generation of flavonolignan (regio-) congeners, such as isosilybin, silychristin, silydianin, and some other minor flavonolignans. Nevertheless, the outcome of these reactions is also influenced by factors such as thermodynamics, stereoinduction, and many others, therefore all flavonolignan diastereomers occur in nature in non-equimolar (often varying) pairs: typically silybin A/B ca. 3–4.5: –6.5, isosilybin A/B ca. 4:1, silychristin A/B ca. 95:5; only silydianin occurs as a single stereomer.

All basic silybin congeners are isobaric (C_25_H_22_O_10_; M.W. 482.44 g/mol); respective 2,3-dehydroderivatives lack two hydrogen atoms (C_25_H_20_O_10_; M.W. 480.42 g/mol).

Martinelli et al. [8] analyzed 200 chemotypes of *S. marianum* (L.) Gaertn. var. *purple* and inferred from the content of the A/B diastereomers that chemotype B (most abundant) has the complete silymarin biosynthetic pathway, whereas chemotype A (presumably a natural mutant) produces a high proportion of silychristin but no silydianin. Chemotype A produces ca. 10× higher amount of silybin A and B than chemotype B. There exists also a rare chemotype C that seems to be a hybrid between A and B. Interestingly, in addition to producing a higher proportion of silydianin, chemotype B also produces double the amount of isosilybin B compared to chemotypes A and C [33]. Isosilybin B generally occurs in silymarin in quite low proportions; however, it exhibited very interesting biological activities [34,35]—see Section 4.4.

2,3-Dehydroflavonolignans form a group of relatively minor flavonolignans whose origin is still somewhat unclear but which are recently gaining more and more attention due to their interesting biological effects. The hydroxyl moiety at the C-3 of flavonolignans is susceptible to oxidation via enolization, especially under alkaline conditions [36]. The oxidants such as O_2_ [37] or I_2_ [38] yield 2,3-dehydroflavonolignans with a stable enol structure. These compounds were formerly considered to be artifacts arising from the oxidation of the parent flavonolignans. Although it was recently found that these dehydroflavonolignans occur in silymarin [10,39], it remains unclear whether they originate directly from the plant material or whether they are artefacts produced during silymarin preparation and/or storage. Presumably, they originate from the parent natural flavonolignans, as they also occur as pairs of stereomers in a proportion similar to the parent flavonoids. However, two chiral centers are removed due to the new 2,3-double bond. The resulting dehydroflavonolignans are thus not diastereomers but enantiomers, therefore they can only be separated with a chiral column. Recently, the first analytical separation of 2,3-dehydrosilybin A and B was achieved using a chiral Lux 3 Cellulose-4 column, allowing an even more accurate description of silymarin composition [10].

### 2.3. Separation of Flavonolignan Diastereomers

Although the separation of the diastereomers does not require chiral methods, it is not easy at all. It is analogous to the difficult separation of the stereomers of carbohydrates—glucose and galactose—which differ only in the position of a single hydroxyl (equatorial vs. axial, respectively), yet their chemical and biological properties are radically different. The chromatographic behavior of flavonolignan diastereomers is so similar that they were originally considered to be a single compound [20,40]. Silybin only yields mixed (equimolar) crystals of silybin A and B [41]; the crystallization of isolated diastereomers failed. This was the reason why most authors wrongly assumed that the silybin stereomers in silymarin occur in equimolar proportions. It was not until 1977 that Tittel and Wagner [42] observed the signal of silybin as two peaks in HPLC; two years later Arnone et al. [43] described for the first time the two diastereomers of silybin, although not knowing their absolute configuration. Nevertheless, it was possible to infer from the ^1^H NMR (of mixed silybin) coupling constants of C-10-C-11 that both diastereomers have a *trans*-configuration at this moiety. Finally, Kim et al. [21] determined the absolute configuration of silybin A (2*R*,3*R*,10*R*,11*R*) and silybin B (2*R*,3*R*,10*S*,11*S*) (Figure 2) using a comparison of ECD spectra of isolated diastereomers with that of the model compound 3-methyl-2-phenyl-1,4-benzodioxane. However, in this publication [21], the absolute configuration was given wrongly in the text and this error was amended later in the corrigendum [21]; nevertheless the respective silybin structures were drawn correctly.

Since the discovery of silybin isomerism, analogous research continued in other silybin congeners. Isosilybin structure [43] and the absolute configurations of both stereomers A and B were established by Lee and Liu [24], who succeeded in an X-ray analysis of the crystal of isosilybin A, and, independently, by Kim et al. [21]. Later, isosilybin A absolute configuration was confirmed by X-ray crystallography of the heavy atom analogue 7-(4-bromobenzoyl)isosilybin A [44]. For a long time, silychristin was thought to occur in nature as a single diastereomer (now silychristin A) [45] until silychristin B was isolated as a minor component in silymarin, which originated in New Zealand [46]. Silychristin B tends to co-elute with silydianin in reversed-phase columns [47], therefore it remained “invisible“ until advanced analytical methods established that both silychristin stereomers occur in most silymarin preparations, though in various proportions [10,11]. Silydianin, which can be easily crystallized [48], seems to be probably the only major silybin congener that exists in nature as a single diastereomer [49,50].

The analytical separation of silybin stereomers was accomplished by numerous authors (vide infra). However, (semi)preparatory separation of the respective diastereomers was not achieved until 1997, mostly due to their very similar chromatographic behavior. A series of glycosides from the mixed natural silybin at the position C-23 was prepared [51]. During the isolation of the respective glycosides in their acetylated forms, the authors observed a fuzzy splitting of the product spot on TLC. After optimizing the chromatographic conditions, gram amounts of silybin A and silybin B 23-β-*O*-glucopyranosides and the respective 23-β-*O*-galactopyranosides were obtained via separation of their peracetates by conventional flash chromatography on silica gel. The bulky and chiral carbohydrate moiety attached in the proximity of the pivotal chiral centers clearly acted as a “chiral handle” to enable the separation. The respective glycosides were then hydrolyzed under acidic conditions to yield pure silybin A and B. The NMR spectra and optical rotations of both optically pure silybin diastereomers were then recorded for the first time. It is important to note that the authors who later established the absolute configuration of silybin A and B [21,24] reversed the A-B nomenclature (see Section 2.1.); therefore, what was referred to as silybin A and B in this investigation [51], are now correctly referred to as silybin B and A, respectively. An easy method for authenticating the respective diastereomer—without the use of standards—is optical rotation: the [α]_D_ of silybin A is always considerably higher than that of silybin B, while natural silybin falls between the two [6].

The above method was optimized to afford multigram amounts of both optically pure silybin diastereomers [52] when the respective silybin 23-β-*O*-galactopyranosides (prepared by catalysis with BF_3_.Me_2_O from silybin and peracetylated galactose, chromatographic separation of the respective peracetylated glycosides followed by Zemplén deacetylation) were mildly hydrolyzed using the β-galactosidase from *Aspergillus oryzae*. Later, it was found that, in the reactions catalyzed by BF_3_.Me_2_O (Lewis acid), the isomerization of silybin at position C-10-C-11 and C-2-C-3 gives small amounts of the respective *cis*-2,3 and/or *cis*-10,11 silybins [25]. These *cis*-silybins are difficult to separate from the natural *trans*-silybin. Due to the large differences in the optical rotation of the *cis*- and *trans*-silybins, even small amounts of the former strongly influence the final [α]_D_ value of the silybins prepared by the above method [6,25].

Pure silybins A and B were obtained directly from natural material in amounts of a few milligrams by repeated preparative reversed-phase HPLC (Ref. [24]—yields not given), (Ref. [21]—ca 5 mg of each diastereomer) in an amount sufficient for spectral measurements to determine their absolute configuration. This method was later scaled up and optimized by Graf et al. [22], who obtained the corresponding gram-scale optically pure flavonolignans by repeated cascades of flash chromatography on silica gel followed by preparative HPLC over repeated injections (approximately 150×), although this took several months. This method later allowed isolation of two new minor flavonoids, having *meta*-position of substituents at the E-ring, e.g., isosilybin C and D [53].

Di Fabio et al. [54] developed a preparatory HPLC method that enabled them to obtain ca. 300 mg of silybin A and B per day. It is clear that this HPLC methodology is suitable for obtaining at most tens to hundreds of milligrams of the respective diastereomers, as it is time-consuming and laborious. Moreover, hi-tech instrumentation is required, not to mention large amounts of HPLC-grade solvents.

In addition to the chromatographic methods, there is another way to obtain pure silybin B. Multiple recrystallizations from methanol, EtOH, acetone, or ethyl acetate of natural silybin (not the crystalline one, which is an equimolar mix of A/B) lead to the enrichment of mother liquors with silybin B. This is based on the fact that silybin B always predominates in natural silybin, which forms equimolar crystals of silybin A and B [41]. This method has never been published in detail but there are anecdotal reports on this method from experts working with silybin [6,55].

In summary, the above methods are unsuitable for obtaining larger amounts of pure silybin diastereomers due to the need for derivatization (with the inherent risk of compound degradation/alteration) or high demands on time and labor.

**Figure 3:**
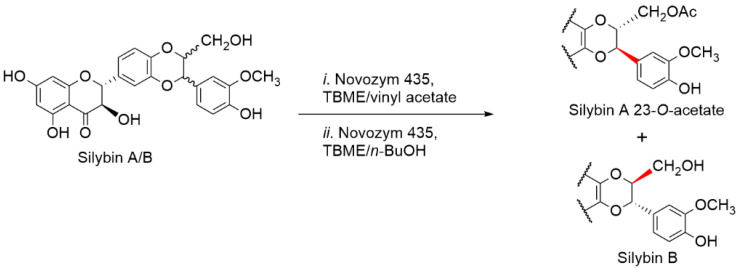
Chiral separation of natural silybin with immobilized lipase B from *C. antarctica* (Novozym^®^ 435), TBME—*tert*-butyl methyl ether. Silybin B and silybin A 23-*O*-acetate are separated by conventional flash chromatography; while pure silybin B is obtained directly, silybin A is then deacetylated with Novozym^®^ 435 under hydrolytic conditions [58].

Silybin diastereomers differ in the chirality adjacent to the primary alcoholic group at C-23. Enzymatic discrimination using lipases proved to be very efficient at solving analogous problems with high selectivity, high yields, mild conditions, and scalability [56]. We tested a large panel of hydrolases (lipases, esterases, and proteases) for the separation of silybin A and B by selective transesterification or by stereoselective alcoholysis at the primary alcoholic group at C-23, which is in close proximity to the central chiral center. *Candida antarctica* lipase B proved to be the most suitable enzyme for the preparative production of silybin A and B [57]. This enzymatic discrimination allowed for the production of gram quantities of the optically pure substances within a few days; moreover, this method is robust and easily scalable to tens of grams. It was optimized and scaled up by Gažák et al. [58] using commercial immobilized *C. antarctica* lipase B (Novozym435). This enzyme catalyzes the regioselective acetylation of natural silybin and the subsequent diastereoselective alcoholysis of the acetylated products to silybin B and 23-*O*-acetylsilybin A, which are easily separated by silica gel chromatography (Figure 3). The optimized method using nontoxic and inexpensive commercially available chemicals (no Lewis acids) and the reusable immobilized lipase was demonstrated at 30-g scale. Both silybin A and B were obtained in diastereomeric excess (*d.e.*) of more than 99%. This method opened the way to the synthesis of new optically pure silybin derivatives and to advanced biological experiments focusing on the molecular targets of the two silybins.

An analogous method using the enzyme-catalyzed acylation/deacylation of C-23 hydroxyl was also optimized for the separation of isosilybin diastereomers at a multigram scale [55]. It was based on the aforementioned enzymatic method followed by repetitive crystallizations. This procedure yielded isosilybin A at over 96% *d.e.* in good yields. Isolation of isosilybin B by this method is more difficult. This is mainly due to the very low percentage of isosilybin B in natural silymarin (typically about 4%).

The above enzymatic protocols were also tested with silychristin A/B. However, none of the tested lipases [59] was suitable for kinetic separation of silychristin A and silychristin B, so only chromatographic methods can be considered [46].

Most of the above procedures require silica gel chromatography or reversed-phase HPLC. Chromatography of polar compounds (phenols) on silica gel suffers from significant losses as some of the analyte is irreversibly bound to the matrix. The scalability of the HPLC methods is often limited. Using gel filtration of isobaric compounds such as silybin congeners could be considered unorthodox, but eventually this method proved to be very useful [60]. The separation of silymarin on Sephadex LH-20 in methanol enabled silydianin devoid of silychristin B (inseparable by HPLC) and silychristin A to be isolated. Moreover, significant amounts of isosilybin AB can be obtained. Even though this method is unable to separate the respective diastereomers, it is a suitable pre-purification step, as it has a high recovery rate. It was tested at a preparatory scale (using an 800 × 50 mm column); a single load of ca. 4 g crude silymarin was separable within one day. The column is reusable ca. 10× and the matrix is fully recyclable.

### 2.4. Analytical Methods for Flavonolignan Diastereomer Determination

A plethora of methods for the analysis of silymarin exist, most of them being based on HPLC with UV, electrochemical, or MS detection. Here we will only evaluate the state-of-the-art methods that discriminate stereomers of silymarin flavonolignans. A complete survey of methods of silymarin analysis is included in a recent review dealing with silymarin composition [9]; another comprehensive review of this analytics covered the literature up to 2015 [61].

The first HPLC separation of silybin diastereomers was probably achieved by Tittel and Wagner [42], who used a -Bondapak reversed-phase column with isocratic elution with H_2_O/MeOH 6:4 containing 5% acetic acid. Later [62], this quantitative method was used for routine analysis of silymarin components in crude extracts and the corresponding preparations. Virtually all subsequent HPLC methods succeeded in separating all major silymarin flavonolignans, including silybin A and B; typically, silybin A eluted first. Considerable improvements were achieved in the 1990s when gra-dient elution was employed—still using a similar setup, e.g., an RP18 column and an acidified mixture of H2O/MeOH as eluent [63]. Nevertheless, these methods—despite separation of silybin A/B and isosilybin A/B—barely reached baseline separation. In 1992 a pioneering study was published by Weyhenmeyer et al. [64], who demonstrated for the first time different pharmacokinetic profiles of silybin A and B in plasma (see Section 3); this would not be possible without the robust and validated HPLC analytical method used [65]. Later the same group [66] published a sophisticated method for the determination of free and total silybin diastereomers in plasma using combined UV and electrochemical detection. In 2007 Hoh et al. [67] developed reliable HPLC-UV and LC-MS analyses with limits of detection of 1–2 ng/mL for each silybin diastereomer.

Monolithic columns allowed the same or even better resolution, but the speed of analysis was improved significantly [68,69]. These columns exhibit a higher selectivity than the particle-packed columns in terms of the selectivity factor (1.21 vs. 1.12, respectively) in the analysis of silybin A and silybin B, and allowed approximately half the run time together with decreasing back pressure [69]. Both types were typical reversed-phase C_18_ columns. The silymarin components were also analyzed with C_30_ columns, but with retention times up to 100 min. The more polar C_8_ columns did not allow for the separation of the silybin diastereomers [70]. Rapid separation of taxifolin, silychristin, silydianin, silybin A, silybin B, isosilybin A and isosilybin B was achieved with a core-shell pentafluorophenyl stationary phase by a UHPLC technique [71]. A very detailed overview of the mobile phases used in silymarin HPLC analysis (typically a combination of acetonitrile and/or methanol with water under acidic conditions) is provided in a review by Chambers et al. [9].

In the HPLC analysis of silymarin, typically UV or better PDA detectors are used. The extinction coefficients of the flavonolignans are similar, but not identical (differing by ca. 15%), which can be directly inferred from the different calibration curves for silybin A and B [10]. UV spectra of 2,3-dehydroflavonolignans differ substantially from the parent flavonolignans; therefore, separate calibrations need to be employed for these two types of compounds, which are often present together in most silymarin preparations.

LC-MS methods appeared at the beginning of this millennium; probably the first comprehensive work on silymarin analysis by LC-MS was published by Khan et al. [72]. The MS discrimination of flavonolignans proved difficult due to their isobaric nature. Kuki et al. [73] optimized the fragmentation energies to reach the best detection of common fragments and hence stereomers. This method was also used in the MS-MS mode [74]. Nevertheless, this was not a universal method, due to a need for advanced MS analyzers (Q-TOF, IT-TOF, IT, SQ) with various instrument setups. Therefore, a specific method validation with authentic standards is mandatory.

In 2020, two very efficient analytical methods were published that solved most of the existing problems in silymarin analysis. The first used UHPLC coupled with drift tube ion mobility spectrometry and quadrupole TOF MS [11]. A total of 11 silymarin compounds were unambiguously identified (taxifolin, isosilychristin, silychristins A and B, silydianin, silybins A and B, 2,3-*cis*-silybin B, isosilybins A and B, and 2,3-dehydrosilybin) and, in addition, five unknown species were found-all with baseline separation within ca. 14 min. The respective compounds were characterized in terms of exact mass, retention time, drift time, fragmentation spectra and collision cross section. Quantification based on ion mobility data showed significantly lower detection limits, an extended linear range and complete separation of interferences from the compounds of interest compared to the traditional approach based on the analysis of LC-Q-TOF data. However, this method, which is dependent on sophisticated hi-tech and very expensive analytical instrumentation, was able to detect previously undescribed isomers (probably enantiomers) that were not even found by detailed NMR analyses—mainly thanks to DTIM separation, which is integrated into a UHPLC and QTOF-MS platform as a third dimension of separation [11]. Nevertheless, the problem of enantiomer separation, which are separable only with chiral devices, remains.

The problem of enantiomer separation in silymarin analysis was solved using another recent method [10]. Here, the first analytical baseline separation of the enantiomers of 2,3-dehydrosilybin A and B was achieved using a Lux 3μ Cellulose-4 chiral column. Virtually all modern isocratic HPLC methods for the separation of silymarin components (for a review see [9,61]) are now rapid and reliable, but allow only partial separation of silychristin B and silydianin, and the peaks with higher retention times are blurred or coelute [47]. Additionally, the separation of all three isomers-isosilychristin, silychristin A, and silychristin B-was not achieved in a single analysis [21]. Recently, the new method enabling the separation and quantification of all known compounds of silymarin—including their respective diastereomers—was published [10]. All the components were found to have different calibration curves—even diastereomers of silybin and isosilybin differ substantially—which implies a difference in their extinction coefficients. Therefore, a specific calibration curve was used for each particular analyte, including the respective diastereomers. Virtually all the previous HPLC methods used a single calibration curve (typically for silybin) for all components, which now seems to be entirely inappropriate and generates mistakes in the respective component quantifications.

For the quantification of 2,3-dehydroflavonolignans, e.g., 2,3-dehydrosilybin, 2,3-dehydrosilychristin, and 2,3-dehydrosilydianin, a different gradient and quantification with ESI-MS in the negative mode was used [10]. The 2,3-dehydroflavonolignan content in silymarin is considerably (ca 10×) lower than the parent compounds; therefore much more sensitive methods need to be used. This method is easily transferable, and it uses standard HPLC-MS instrumentation.

NMR spectral methods cannot be readily used to distinguish flavonolignan diastereomers because their spectra have very similar coupling patterns and chemical shift differences that are far below the usual accuracy level of 0.01 ppm, and numerous peaks of the respective stereomers overlap; there are no clear diagnostic signals for their distinction [21,24,51]. A new spectroscopic method using a 900-MHz NMR spectrometer allows, by means of computer-assisted 1H iterative full-spin analysis (HiFSA), the interpretation of ^1^H NMR data of pure isomers, the generation of their ^1^H fingerprints, and the subsequent analysis of different mixtures of silybin A/B and isosilybin A/B [23]. Most Δδ_H_ values fall in the low ppb range, allowing the identification and quantification of individual flavonolignans, even at 300 MHz. The magnitude of these chemical shift differences clearly demonstrates the need for a third decimal place when reporting NMR data of silybin derivatives. A detailed spectral analysis of silybin A and B, including chiroptic properties, detailed UV, ECD, IR, Raman spectra and corresponding molecular modeling of the theoretical spectra based on density functional theory calculations has been published by Solís-Gómez et al. [75]. This study provides a valuable aid in the discrimination of flavonolignan diastereomers. From the practical point of view, it is obvious that recent advanced HPLC techniques using standard equipment will be further broadly used in silymarin analysis.

### 2.5. Chemical and Enzymatic Transformations of Optically Pure Silybin A and B

The isolation, preparatory separation of diastereomers of silybin, and determination of their absolute configuration paved the way to the work with optically pure flavonolignan diastereomers. This enabled an easier structural analysis of products with only a single set of NMR signals of one stereomer, and mostly stimulated the preparation of optically active derivatives for analyzing the molecular interactions of particular compounds. Molecular studies with mixed silybin may have been justified when its pure stereomers were unavailable, but in the light of current knowledge, such studies should be considered obsolete, misleading, or even methodically wrong.

**Figure 4:**
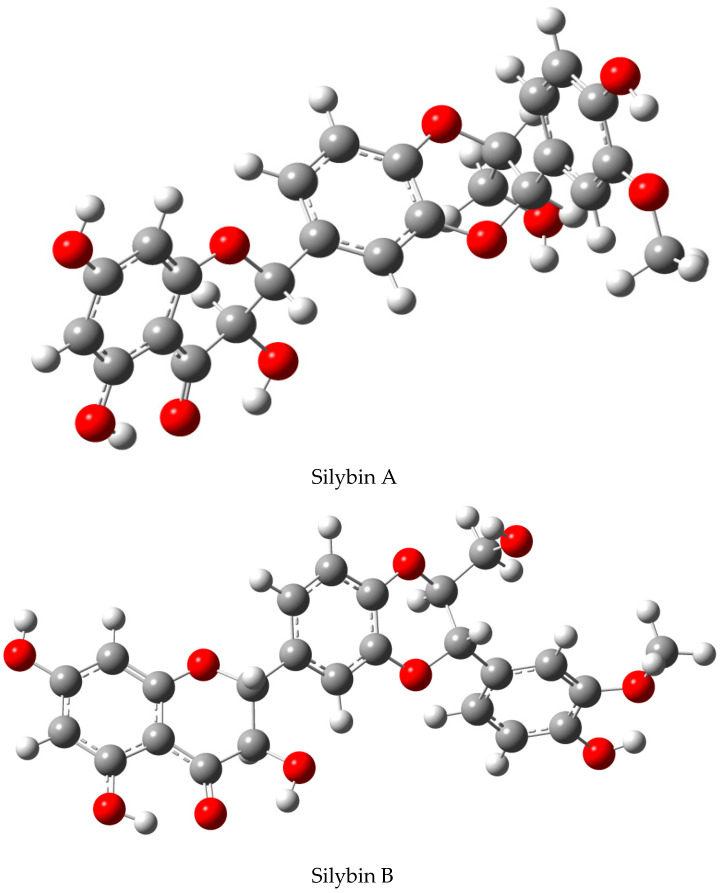
Molecular models of silybin A and silybin B; color code: red—oxygen; grey—carbon; white—hydrogen. Energy minimized structures prepared in program Gaussview https://gaussian.com/gaussview6/ (accessed 20 January 2021).

Simple molecular modeling (energy minimization) clearly shows fundamental structural and steric differences between silybin A and B: Silybin A has a relatively curved structure, resembling a “banana shape”, whereas silybin B is planar (Figure 4). Chemically, both diastereomers react quite similarly, especially when the reaction occurs in an isotropic milieu (e.g., solution without chiral reactants). Nevertheless, in an anisotropic environment, typically with enzymes or other biological systems, each stereomer behaves differently. Most of the chemical modifications published so far have been performed with the mixed silybin A/B, although recently there have been advanced chemical studies using optically pure silybins.

The antioxidant capacity of both silybin diastereomers using in vitro methods seems to be very similar; however, it is not identical, taking into account the experimental error [3,76]. However, this is not the case of complex 3D biological systems where the parameters of both stereomers differ significantly.

Metal complexation (copper and iron) of silybin A and B, 2,3-dehydrosilybin A and B, and silychristin A was studied by Tvrdý et al. [77]. Only 2,3-dehydrosilybin was identified as a strong or moderately active copper and iron chelator, whereas silybin A and B and silychristin A were less strong or inactive chelators. Stereoisomerism in silybin and its 2,3-dehydroderivative did not play a role, possibly due to the distance of the optical centers (C-10, C-11) from the chelating site.

Probably the first transformation study with optically pure silybin A (which is now correctly denoted as silybin B) was published in 1998 [78]. Silybin was glycosylated in excellent yields using cell culture of *Papaver somniferum* to yield silybin-B-7-*O*-β-d-glucopyranoside. The same silybin isomer A (now silybin B) was used for the preparation of potential silybin metabolites β-d-glucuronates using ovine liver UDP-glucuronyl transferase and uridine 5′-diphosphoglucuronic acid as a glycosyl donor. This reaction produced silybin β-glucuronates substituted at positions C-20, C-7, and C-5 at a semi-preparatory scale (tens of mg) in yields of 27, 62, and 2.5%, respectively [79]. These conjugates proved to be instrumental in pharmacological studies (see Section 3). Charrier et al. [80] succeeded in the preparation of optically pure silybin A and B β-d-glucuronides at positions C-7, C-20, and a smaller amount at C-5, at a 100 mg scale. Both optically pure silybins were subjected to biotransformation by submerged culture of *Streptomyces* sp. strain M52104, which produced the above glucuronides in quasi-quantitative yields. Gufford et al. [81] optimized the previously published method [79] and prepared the respective silybin A and B glucuronides at a scale of tens of mg.

Jančová et al. [82] demonstrated a clear stereoselectivity of silybin glucuronidation using a human liver microsomal fraction, human hepatocytes, and twelve human recombinant UDP-glucuronyl transferases. Human liver microsomes preferentially formed 7-*O*-β-d-glucuronides and lower amounts of 20-*O*-β-d-glucuronides. Silybin B was the predominantly glucuronidated diastereomer. This effect was in accordance with the performance of respective UDP-glucuronyl transferases—this will be discussed in detail in Section 3.

Sulfates are other important conjugates of xenobiotics formed in the Phase II of xenobiotic metabolism. The sulfation of silybin seems to be strictly stereoselective. The sulfation of silybin A and B with the recombinant rat liver arylsulfotransferase AstIV demonstrated that only silybin B is sulfated, yielding 20-*O*-silybin B sulfate, while silybin A was entirely resistant to the sulfation [83]. This finding is crucial for ADME studies of silybin diastereomers and is consistent with other studies that have investigated the pharmacokinetics of silybin diastereomers [64,84]. The 20-*O*-silybin A sulfate was obtained by the enzymatic sulfation of optically pure silybins with recombinant aryl sulfotransferase from *Desulfitobacterium hafniense* [85]. This enzyme was able to sulfate both silybins A and B at approximately the same rate. However, upon isosilybin sulfation, a much lower reaction rate was observed for isosilybin B compared to the A stereomer (formation of 20-*O*-isosilybins). Thus, stereodiscrimination takes place here as well.

7,23-Disulfates of both silybin A and B (silybin A-7,23-diyl *bis*(hydrogen sulfate) and silybin B-7,23-diyl *bis*(hydrogen sulfate) were synthesized by the use of a pyridinium-sulfur trioxide complex in dimethylformamide in a 60% yield [86]. These compounds were tested for anti-cancer efficacy, and it was shown that these potential silybin metabolites exhibit a considerably lower anticancer activity than the parent molecules.

The stereoselectivity of lipases towards silybin diastereomers was largely demonstrated by their enzyme-mediated separation (see Section 2.3) [57,58]. Lipases were also used for the regioselective acylation/deacylation of silybin A and B [87]. Silybin has five OH groups, which makes it a challenging target of the protection/deprotection strategy, and regioselective enzymatic methods can be well employed. Here, extensive screening selected the lipase AK that selectively deacetylates pentaacetylsilybins A and B to yield 3,5,20,23-tetra-*O*-acetyl-silybins A and B and 3,20,23-tri-*O*-acetyl-silybins A and B, respectively. These new compounds now serve as stereochemically pure synthones for the selective silybin modifications.

**Figure 5:**
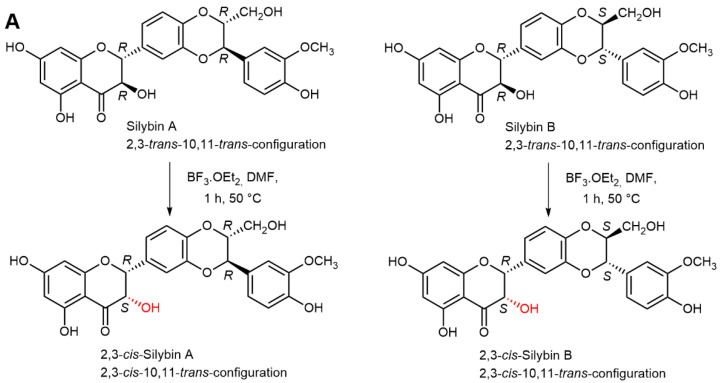

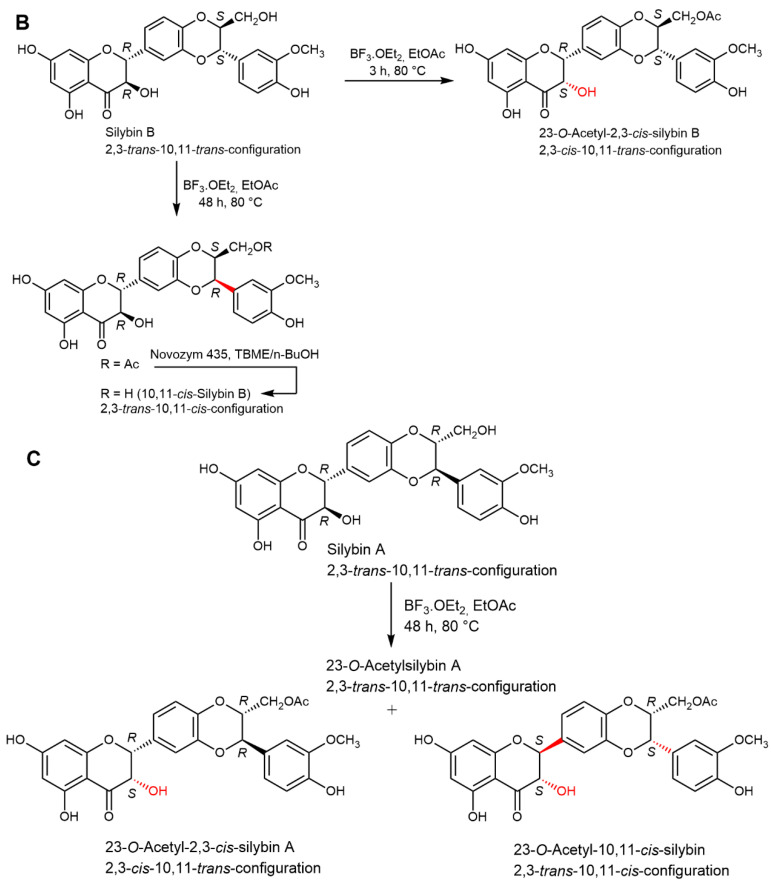
*cis-trans* Isomerizations of the optically pure silybins [25]. (**A**) Silybin A and silybin B isomerizations into respective 2,3-*cis*-isomers (DMF, dimethylformamide). (**B**) Silybin B isomerization in EtOAc (TBME, *tert*-butyl methyl ether). (**C**) Isomerization of silybin A in EtOAc.

The availability of the optically pure silybins enabled us to selectively modify all respective chiral centers at C-2,3 and C-10,11 bearing the *trans*-configuration in natural silybins, which would be impossible with mixed silybin. Using BF_3_.Et_2_O as a catalyst under various conditions, it was possible to prepare and fully characterize 2,3-*cis*-isomers by inversion of the C-3 OH, namely (2*R*,3*S*,10*R,*11*R*)**-silybin (2,3-*cis*-silybin A) and (2*R*,3*S*,10*S,*11*S*)**-silybin (2,3-*cis*-silybin B) (Figure 5A). Under different conditions, starting from silybin B and then configuration inversion at C-11, (2*R*,3*R*,10*S,*11*R*)**-silybin (10,11-*cis*-silybin B) was formed (Figure 5B). However, silybin A gave two products under the same conditions: (2*R*,3*S*,10*R,*11*R*)**-silybin (2,3-*cis*-silybin A) and the product of triple inversion of the configuration at C-2, C-3, and C-11, i.e., (2*S*,3*S*,10*R,*11*S*)-silybin [25] (Figure 5C). Both the 2,3- and 10,11-*trans*-configured flavonolignans seem to be more stable than the respective *cis*-isomers, which tend to (partially) isomerize back into the *trans*-configuration under acidic or alkaline conditions. Some silybin *cis*-isomers are presumably present in various silymarin preparation, as was recently demonstrated by Fenclová et al. [11].

The only practically feasible way to prepare optically pure 2,3-dehydroderivatives of flavonolignans (the introduction of a double bond into the C-ring), e.g., 2,3-dehydrosilybin A and B is by oxidation of the respective silybins A and B [37]. As recently demonstrated by Petrásková et al. [10], 2,3-dehydroflavonolignans are enantiomers, and therefore they are only separable by chiral columns. Gažák et al. [36] prepared the first pure 2,3-dehydrosilybins A and B by oxidating the respective silybins with oxygen under alkaline conditions. This method—based on the same principle—was later slightly optimized by Di Fabio et al. [54].

**Figure 6:**
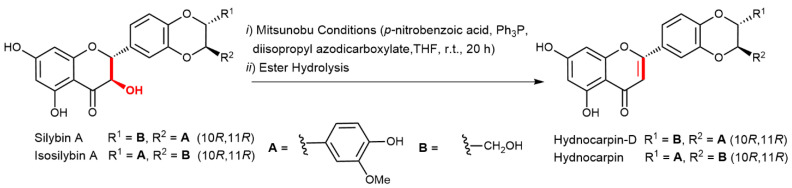
Example of hydnocarpin preparation from flavonolignans using Mitsunobu conditions [89].

Another skeletal modification of silybin and some other flavonolignans accompanied with the introduction of the double bond at C-2,3 is 2,3-dehydration, which yields hydnocarpins; flavonolignans formally derived from luteolin (wheras major silymarin flavonolignans are derived from taxifolin) [88]. Here, the use of optically pure flavonolignans is essential to obtain particular optically active hydnocarpins, which retain the C-10,11-configuration from the parental molecules; the chiral centers at C-2,3 are abolished by a new double bond. Dehydration of the respective flavonolignans under Mitsunobu conditions (Figure 6) gave optically pure hydnocarpin isomers, e.g., (10*R*,11*R*)-hydnocarpine from isosilybin A, (10*R*,11*R*)-hydnocarpin D from silybin A, (10*S*,11*S*)-hydnocarpin D from silybin B, and isohydnocarpin from silychristin A [89]. An alternative four-step reaction sequence to obtain the respective hydnocarpins from optically pure flavonolignans was performed by Vimberg et al. [90]. In the first step, regioselective formylation of the C-3 OH of the respective 23-*O*-acetylated flavonolignan was carried out using the Vilsmeier- Haack reagent ((COCl)_2_ in dimethylformamide); subsequent formic acid elimination by triethylamine then yields optically pure hydnocarpin isomers, e.g., (10*R*,11*R*)-hydnocarpin, (10*R*,11*R*)-hydnocarpin D, (10*S*,11*S*)-hydnocarpin D, and isohydnocarpin from silychristin A. These compounds were found to be potent inhibitors of *Staphylococcus aureus* biofilm formation, where (10*S*,11*S*)-hydnocarpin D was the most active. This compound also increases the susceptibility of antibiotic-resistant *S. aureus* towards chinolone antibiotics, probably by the inhibition of MDR transporters [90]. Here, the stereochemistry at C-10,11 is absolutely crucial for the respective biological activity.

A panel of C-23 derivatives of silybin A and B (sulfate, azide, phosphodiester, and amine) were prepared [91] to investigate the antioxidant behavior of both series of A and B derivatives with a standard DPPH assay. The antioxidant activities of particular A/B pairs of derivatives were practically identical within the margin of experimental error. This is not surprising as silybin A and B behave very similarly in isotropic systems (in methanol solution) [76,92] and moreover, the C-23 silybin moiety is virtually redox-inactive [93,94]; the most radical/redox-active moieties are the C-7 and C-20 OH groups.

Even simple derivatization, such as methylation, can significantly alter the biological activity of flavonolignans. Agarwal et al. [86], among others, prepared 7-*O*-methylsilybin A and B for anticancer studies by direct base-catalyzed methylation of the respective silybins [95]. When testing anti-cancer efficacy with human bladder cancer cells HTB9, colon cancer cells HCT116 and prostate cancer cells PC3, they found that these two derivatives consistently exhibited better anti-cancer efficacy than si-lybin, but no appreciable difference was found between the A and B derivatives [86] (see also Section 4.4). Althagafy et al. [96] prepared a series of 7-*O*-methyl derivatives of optically pure flavonolignans (silybin A + B, isosilybin A + B, silychristin A, isosilychristin and silydianin) and Sy-Cordero et al. [97] prepared a series of mono- and oligo-methylated derivatives of silybin B—both authors used a published methodology previously applied to mixed silybin [95]. All of the above derivatives were extensively tested for biological activities (see Section 4).

Hurtová et al. [98] demonstrated a novel, mild and selective method for the bromination of polyphenols including pure silybin A and B using α,β-dibromohydrocinnamic acid in the presence of a base. Silybin A and B yielded 6,8-dibromosilybin A and B, respectively, in similar yields and with the same selectivity—the reaction proceeded in a homogeneous solution. The reaction was highly selective using Cs_2_CO_3_ at 40 °C yielding only 6-bromosilybin A and B.

The conjugation of silybin with some other lead structures has become quite popular recently, and there are a handful of examples where the authors used optically pure silybins and performed decent molecular studies. Probably the first such synthetic conjugation study using optically pure silybin A and B was the preparation of 7-*O*-galloylsilybin A and B for studies on antiangiogenic activity [99]. This derivative was prepared from both silybins by a reaction with 3,4,5-tri-*O*-benzylgalloylchloride, followed by hydrogenolytic debenzylation with an overall yield of over 70%. Other galloyl derivatives substituted at C-3, C-20, and C-23 were prepared from mixed silybin. The most antiangiogenic-active derivative was the 7-*O*-galloylsilybin B (twice as active as the respective A isomer).

Schramm et al. [100] prepared 7-*O*-feruloylsilybin A and B by the regioselective acylation of silybin A and B with the respective acyl chloride (prepared in situ). The respective optically pure esters exhibited a significant difference in both neuroprotection and anti-aggregation (amyloid and τ-protein aggregation) capacities—in both assays, 7-*O*-feruloylsilybin B was more active than the respective A derivative. This is in accordance with the finding of Sciacca et al. [101] that silybin B fully abolished the aggregation process of Aβ40 amyloid, whereas silybin A only slowed it down.

**Figure 7:**
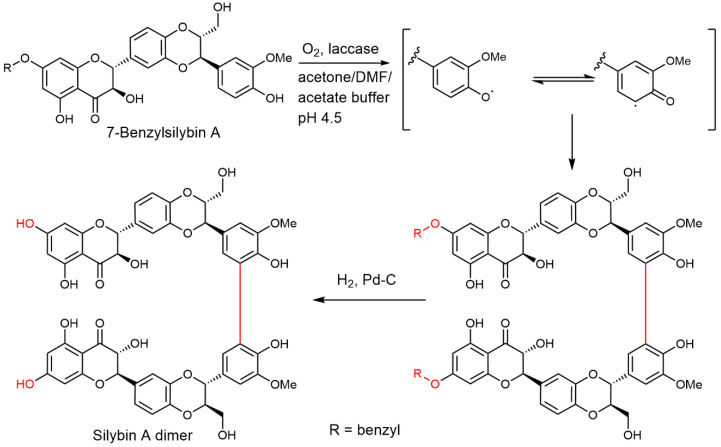
Laccase catalyzed dimerization of silybin A [92].

The use of pure silybin diastereomers proved to be instrumental in the enzymatic synthesis of silybin dimers [92]. Silybin A or B were benzylated at C-7, and the respective dimerization of the protected silybins was catalyzed by the laccase from *Trametes versicolor*. After debenzylation (Pd/C, H_2_), the respective C-21-C-21′ dimers were isolated (Figure 7). The use of optically pure silybins enabled the unequivocal determination of relatively complex dimeric structures; with mixed silybin, a very complex and inseparable mixture of stereomers would be formed. Silybin dimers exhibited a considerably higher (5×) antioxidant capacity than the respective monomers.

**Figure 8:**
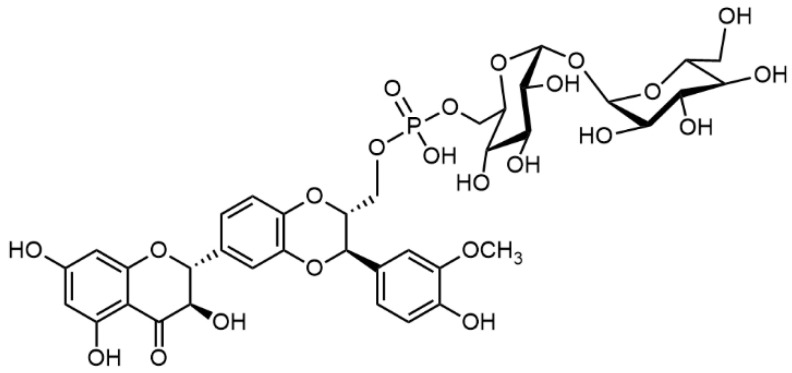
Structure of silybin A conjugate with trehalose linked via phosphate diester bond [102].

The above findings prompted the group of García-Vinñuales et al. [102] to prepare other C-23 conjugates of silybin A and B with trehalose linked via a phosphate diester bond (Figure 8) by coupling reactions between the respective silybin-23-phosphoramidite and the appropriately protected trehalose, catalyzed by 4,5-dicyanoimidazole. Here again, a better activity in terms of inhibiting the aggregation of amyloid β peptide was found in the respective silybin B derivative. Unfortunately, the authors did not consider transport over the hemato-encephalitic barrier, which would be presumably impermeable for such a big molecule.

## 3. Pharmacokinetics of Silybin with Regard to Its Stereochemistry

There are over 200 papers dealing with various pharmacological aspects of silymarin ADME (absorption, distribution, metabolism, and excretion), including that of silybin (for reviews, see [103,104,105]).

The first stereospecific assay of silybin published by Mascher et al. [65] enabled the pharmacokinetic study of silybin diastereomers in human volunteers [64]. Six human male volunteers were given various doses of silymarin (Legalon 140, Madaus, Cologne, Germany) up to 254 mg/dose. Plasma concentrations of conjugated and unconjugated silybin diastereomers were monitored for 16 h after the administration. The authors clearly established that the diastereomers had fundamentally different pharmacokinetic profiles. At that time, the authors referred to the silybin diastereomers as “isomer 1 and 2” without knowing their absolute configuration. Based on the order of elution of the respective peaks in the HPLC profile [10,14,24], it can now be deduced that isomer 1 is silybin A, and isomer 2 is silybin B. The concentration of free isomer 1 (A) was approximately 4× higher than that of isomer 2 (B) after 4 h. Nevertheless, the concentration of total isomer 2 (B)—after deconjugation—was ca. 3× higher than that of isomer 1 (A). Apparently, silybin B (isomer 2) is conjugated much more quickly than silybin A (isomer 1) and this is also reflected in the average area under the curve (AUC) in all doses being ca. 3× higher for silybin B than for silybin A. This observation was later confirmed in several other studies, including one using pure silybin diastereomers administered to rats [84]. Later, both diastereomers were also shown to affect the metabolism of the other when subjected to biotransformation as a mixture [106].

An analogous experiment was later performed by Wen et al. [107]; they monitored the metabolism of all major flavonolignans in plasma samples of three healthy volunteers after a single oral dose of 600 mg of standardized milk thistle extracts. They identified silybin B glucuronide(s) as a major silybin metabolite, whereas the rate of silybin A/B glucuronidation was ca. 1:3. With isosilybin, the A diastereomer was found to be ca. 2-fold more glucuronidated than its B counterpart. Here, however, it should be noted that the B isomer occurs in silymarin in a ca. 4-fold lower amount than the A isomer, and since the volunteers were given nonprocessed silymarin, this proportion could influence the occurrence of their metabolites found in plasma. They also found that silybin sulfation is considerably lower than its glucuronidation.

**Figure 9:**
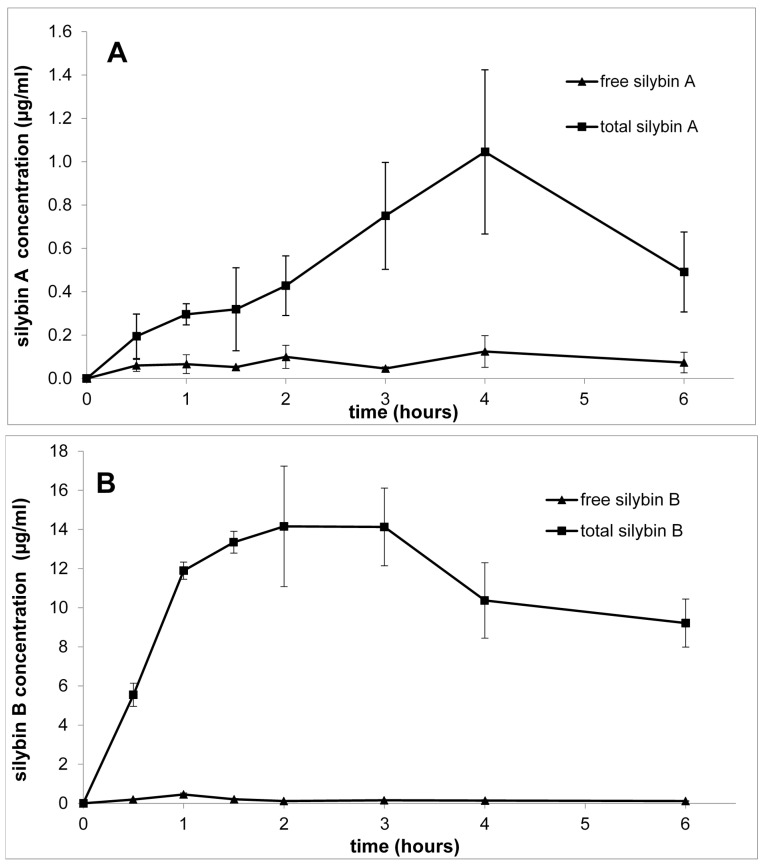
(**A**) Plasma concentration-time profile of free (unconjugated) and total silybin A in rats; (**B**) free and total silybin B after gastric administration of a single dose of 200 mg-kg^−1^ body weight (mean of three animals, error bars—standard deviation) [84].

There is probably only a single study so far that has investigated the in vivo pharmacokinetics of isolated silybin A and B administered separately in rats [84]. Such a study required ca. 30 g of each pure diastereomer, which was enabled by the advanced separation methods developed earlier [58]. Both silybins were administered by intragastric gavage as a single dose of 200 mg/kg b.w., and at the chosen times (0.5–6 h) plasma was analyzed for silybin and its metabolites. The acquired data demonstrate that both silybins are quickly absorbed and eliminated with a short T_1/2_—(ca 2–3 h), and they have a substantially different metabolic fate (Figure 9). Silybin B is sulfated and glucuronylated much faster and to a greater extent than silybin A. These results show that silybin B (C_max_ = 14.50 μg/mL (0.03 μM), T_max_ = 2.6 h, T_1/2_ = 2.9 h; total silybin B) was absorbed faster and in a substantially higher amount (over 10×) than silybin A (C_max_ = 1.05 μg/mL (0.0024 μM), T_max_ = 3.9 h, T_1/2_ = 2.2 h; total silybin A). The oral bioavailability of silybin B was estimated to be 0.3%, while that of silybin A was only 0.03%, which is 10 times lower. These results are in accordance with human studies with silymarin [64,66]. Later human pharmacokinetic analyses, such as that by Zhu et al. [108], which monitored all major silymarin flavonolignans separately in much detail, confirmed the quick biotransformation of silybin B and the highly different bioavailability of the respective diastereomers. The different profiles of both silybin diastereomers must be caused mostly by their different biotransformation rates.

The phase I biotransformation (functionalization) of silybin accounts for ca. 1–2% of the total parent substance. Prominent enzymes of phase I are cytochromes P-450 (CYPs). Gunaratna and Zhang [109] demonstrated the formation of demethylated silybin (presumably 19-*nor* silybin) as a major metabolite and mono- and dihydroxy silybins as minor metabolites after incubation with human liver microsomes. Even though they used racemic silybin, their HPLC analysis demonstrated that both demethylated silybins (separated in HPLC profile) are formed at a comparable rate. Jančová et al. [110] later confirmed this result and showed that isoenzyme CYP 2C8 is responsible for the reaction leading to *O*-demethylated silybin.

The formation of C-19 *O*-demethylated silybins A and B and also C-19 *O*-demethylated isosilybins A and B was later demonstrated by Zhang et al. [111] using transformation by the anaerobic bacterium *Eubacterium limosum* isolated from the human intestine. They succeeded in isolating all the respective C-19 *O*-demethyl derivatives and confirmed their structures (MS, NMR, ECD).

Vrba et al. [112] performed metabolic transformations of a series of flavonolignans with primary cultures of human hepatocytes and recombinant human cytochromes P450 (CYPs 1A2, 2A6, 2B6, 2C8, 2C9, 2C19, 2D6, 2E1, and 3A4). In addition to demethylations and hydroxylations, hydrogenation and dehydrogenation reactions were also observed in isosilybins A and B. These findings were later confirmed by Chen et al. [113], who tested all the individual principal flavonolignans with recombinant human cytochromes P450 and established that CYP 3A4 is responsible for the majority of the biotransformation of all the compounds. They determined the major site of hydroxylation to be ring A in all flavonolignans.

**Figure 10:**
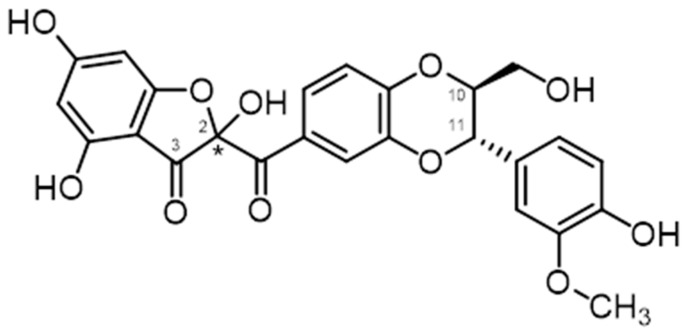
Structure of silybin B metabolite 2,4,6-trihydroxy-2-(3-(4-hydroxy-3-methoxyphenyl)-2-(hydroxymethyl)-2,3-dihydrobenzo[1,4]dioxine-6-carbonyl)benzofuran-3-(2*H*)-one [84].

The oxidation of silybin typically yields 2,3-dehydrosilybin [36], which has not been identified in plasma after silybin administration so far [84]. Another oxidative metabolite 2,4,6-trihydroxy-2-(3-(4-hydroxy-3-methoxyphenyl)-2-(hydroxymethyl)-2,3-dihydrobenzo-[1,4]dioxine-6-carbonyl)-benzofuran-3(2*H*)-one (Figure 10) has been proposed to be a result of an oxidative attack on silybin. This minor, however quite unique, metabolite was indeed observed in the plasma after silybin B administration, and its structure was confirmed by comparison with an authentic sample prepared by chemical synthesis [84].

Conjugation reactions of phase II are the major metabolic reactions of flavonolignans. So far, methylations, glucuronidations, sulfations, and glutathione conjugation were observed.

Methylation is a minor metabolic pathway of silybin, first described by Marhol et al. [84]. After administration of silybin to rats, small amounts of methyl conjugates were found, with 20-*O*-methyl silybin B predominating. In addition, small traces of 5-*O*-methyl-silybin B and 7-*O*-methyl-silybin B were detected in plasma samples. Determination of the structure of methyl derivatives by MS techniques was made possible by the large library of methyl derivatives of silybin previously synthesized [93]. Methylation of silybin A has not been observed previously. Methylation of isosilybin was also observed during incubation with isolated human hepatocytes: Isosilybin B was hydroxylated and methylated, whereas isosilybin A was only converted to a methyl derivative [112]. Isosilybin A was metabolized only by CYP 3A4, which performed either C-19 *O*-demethylation or conversion into mono- and dihydroxylated products. In contrast, isosilybin B was hydroxylated by CYPs 2C8, 2D6 and 3A4 or C-19 *O*-demethylated by the latter two enzymes [112]. It should be noted here that some synthetic silybin B methyl derivatives exhibited some inhibitory activity towards CYP 2C9-mediated (*S*)-warfarin 7-hydroxylation in human liver microsomes—the most potent being 7,20-*O*-dimethyl-silybin B (approximately 50% inhibition at 10 M) [97]. Additionally, 7-*O*-Methylderivatives of all major silymarin flavonolignans were tested for the inhibition of CYP 2C9, where the parent compounds and methyl derivatives gave ambivalent results, except for silydianin and its 7-*O*-methylsilydianin (ca 40% and 10% respectively of the control at 10 μM), CYP 3A4 was stimulated by 7-*O*-methyl silybin A (ca 160% of the control at 10 μM), but other compounds did not exhibit any notable activity, and UDP-glucuronosyltransferase was slightly inhibited by silybin A, but not with its methyl derivative and other compounds [29].

The glutathione (GSH) conjugation of silymarin flavonolignans was described by Chen et al. [113] in isolated silymarin flavonolignans (silybins A and B, isosilybins A and B, silychristins A and B, and silydianin). GSH conjugates of the parent, demethylated, and hydroxylated flavonolignans were identified. GSH conjugates occurred in all flavonolignans both in the ring E and ring A containing a catechol moiety formed previously by hydroxylation (A ring) or demethylation (E-ring) by CYP. The resulting catechols are most likely oxidized to the corresponding electrophilic intermediates *o*-quinones, followed by GSH conjugation.

Obviously, the major conjugation pathway (and in general the major biotransformation pathway) of silymarin flavonolignans is glucuronylation. Glucuronates of pure silybin B (before 2003 denoted as silybin A) were prepared by ovine liver glucuronyltransferase and employed as authentic standards to monitor the site of silybin glucuronylation in humans. It was shown that the main silybin conjugate in humans is 20-β-d-glucuronyl, while the C-7 glucuronate was formed at a lower rate [79].

Han et al. [106] subjected pure silybins A and B to glucuronylation with bovine liver microsomes, and then isolated and structurally characterized (MS, NMR) the respective glucuronates. They demonstrated that silybin B is glucuronylated somewhat faster than silybin A, forming 20-β-d-glucuronate of silybin B as the major product and less than half that amount of the C-7 isomer, whereas silybin A was glucuronylated equally at both the C-7 and C-20 positions. Interestingly, when both silybin A and B were incubated with the microsomes as a mixture, their glucuronylation was slower than when they were incubated separately.

Jančová et al. [82] investigated in detail differences in the regio- and stereoselective glucuronylation of silybin diastereomers using hepatocytes, human liver microsomes, and a series of twelve recombinant UDP-glucuronosyltransferases (UGT 1A1, 1A3, 1A4, 1A6, 1A7, 1A8, 1A9, 1A10, 2B4, 2B7, 2B15, and 2B17); nine subfamilies (UGT1A1, 1A3, 1A6, 1A7, 1A8, 1A9, 1A10, 2B7 and 2B15) were found to be active in the glucuronylation of both silybins. Human UGTs preferentially formed 7-*O*-β-d-glucuronates of both silybin diastereomers and also 20-*O*-β-d-glucuronates. Silybin B was glucuronylated more strongly than the respective A stereomer, with the exception of intestinal UGT1A8, which preferred silybin A. UGTs 1A1 and 1A3 were most potent in the formation of glucuronates, but UGT1A1 was completely unable to synthesize silybin A-20-*O*-β-d-glucuronate. When A and B stereomers were incubated together with UGTs in the mixture, slightly lower glucuronylation was observed for the A stereomer than when both diastereomers were used separately. This fact suggests competition between the two stereomers for glucuronylation, with silybin B clearly having a higher affinity for this enzyme.

Vrba et al. [114] studied the glucuronylation of all silymarin major flavonolignans as optically pure compounds with human hepatocytes, human liver microsomes, or human UDP-glucuronosyltransferases. They fully confirmed the above conclusions and demonstrated that also in other flavonolignans (isosilybin A, B, silychristin A, and silydianin) UGT 1A1 has the highest relative activity towards individual flavonolignans. There is, however, a single exception—isosilybin B, compared to which UGT 1A1 has only 49% relative activity and the most active enzyme in this particular case is UGT 1A8.

All flavonolignans, except for silydianin, undergo glucuronylation in Phase II but they all also form sulfates [107,112], although to a lower extent than glucuronates. The stereodiscrimination of silybins by the respective sulfotransferases is even more profound than UGTs. Recombinant arylsulfotransferase IV from rat liver exclusively sulfated silybin B at C-20 in near quantitative yield (over 5 days), while silybin A remained completely unaffected [83].

A highly detailed analysis of sulfation of silymarin flavonolignan has been recently Vrba et al. [115] using all the major pure flavonolignans. This study investigated the sulfation of individual silymarin flavonoids by the human liver and intestinal cytosols. After the respective incubation, each of the tested flavonolignans yielded at least one sulfated metabolite. The susceptibility of particular flavonoids to sulfation by the intestinal cytosol was as follows: isosilybin A ≈ silychristin A ≈ isosilybin B > silydianin ≈ silybin B ≈ silybin A. Then, a series of recombinant human sulfotransferases (SULTs) were tested (1A1*1, 1A1*2, 1A2, 1A3, 1B1, 1C4, and 1E1) for the sulfation. All the compounds tested yielded exclusively monosulfated products, most of which corresponded to those obtained when the tissue cytosols were used. The most abundant sulfated metabolite products were silybin A 20-*O*-sulfate, silybin B 20-*O*-sulfate, and isosilybin A 20-*O*-sulfate. SULT1E1 produced the highest amounts of silybin A and silybin B sulfates; the second most active enzymes for silybin A and B were SULT1A3 and SULT1C4, but these enzymes mostly produced sulfates other than SULT1E1.

In conclusion, silybin B is generally metabolized (mostly conjugated) to a much larger extent than silybin A. The most active moieties in its molecule for biotransformation are the C-7 and C-20 hydroxy groups for conjugation reactions and C-19 for *O*-demethylation. Analogous differences were observed in biliary excretion in perfused rat livers, e.g., conjugates of silybin B (32%) vs. silybin A (21%) [116]. Silybin B is metabolized significantly faster than silybin A. In rats, the AUC_0__→6h_ value of total silybin B was 20-fold higher than that of silybin A [84]. Therefore, the pharmacokinetic profiles of the two silybin diastereomers are fundamentally different. For ADME studies of silybin, rats are an acceptable model for translation to the human model.

## 4. Specific Biological Effects of Silybin A and B and Other Optically Pure Flavonolignans

There is an indisputable fact that silybin diastereomers behave differently in 3D systems (all biosystems) and often also in an isotropic milieu. Recently, every year some 300 papers dealing with various biological effects of silybin or silymarin are published (source: Web of Science) but only a few of them use isolated diastereomers, and therefore only those few can be considered to investigate molecular effects. Here we will only survey the studies based on pure silybin diastereomers (Table 1).

The first biological investigations of optically pure silybins were limited by their availability to a few research groups and companies that had access to these compounds (although methods for their isolation were published in 1997–2003). Flavonolignan stereomers have been commercially available in pure form since ca. 2010 (among others, e.g., PhytoLab GmbH & Co. KG or Sigma-Aldrich Co.), so now there is no barrier to using pure compounds for molecular studies.

### 4.1. Estrogenic Activity

Many phytochemicals, including flavonoids, elicit agonistic and/or antagonistic effects at the aryl hydrocarbon receptor (AhR) and also interact with estrogen receptors (ER). Seidlova-Wuttke et al. [147] demonstrated that silymarin binds to cytosolic estrogen receptors (model—ovariectomized rats, subcutaneous administration). Interaction with ERα was ruled out in this case. This study was limited to the silymarin extract, and the authors speculated that the compound responsible for this activity was silybin, however, only based on its major content in this preparation. This prompted us to investigate in detail the interactions of the optically pure silymarin components with ER and AhR using in vitro reporter gene assays [117]. We found that neither silymarin nor its components (silybins A and B, dehydrosilybin, isosilybins A and B, silychristin, silydianin, taxifolin, and quercetin) affected the AhR-mediated activity in rat hepatoma cells. No anti-estrogenic effect (a decrease in estradiol (E2) response) of the silymarin constituents was also observed. However, some of the flavonoids tested acted as either partial or complete ER agonists. Silymarin elicited partial ER activation. Silybin B proved to be the compound responsible for the weaker ER-mediated activity (EC_25_ 4.4 μM) of silymarin. Silybin A and other flavonolignans were entirely inactive; only weaker activity was identified in taxifolin and quercetin, as they are minor silymarin components. This result is very important because it has been reported that silybin B is present in the blood mainly in the free, unconjugated form (about 10× higher than silybin A) [64,84]. The estrogenicity of these silymarin components must be considered as a potential (side) effect in their application.

### 4.2. Antidiabetic and Anticholesterolemic Activity

Silymarin acts as a potential hypocholesterolemic agent and significantly improves the plasma lipoprotein profile by increasing the levels of high-density lipoproteins (HDL) and decreasing the levels of VLDL and triacylglycerol [148]. This effect may positively influence liver functions and reduce some pathologies, but it may also be beneficial in the prevention of cardiovascular disease. The transmembrane ATP-binding cassette transporter A1 (ABCA1) in THP-1-derived macrophages has a fundamental role in reverse cholesterol transport by trafficking intracellular cholesterol and phospholipids into lipid-depleted apolipoproteins. Cholesterol export from macrophages or tissues mediated by ABCA1 is considered beneficial for cardiovascular disease prevention. Wang et al. [119] reported that the silymarin components isosilybin A, silybin B, si-lychristin, isosilychristin, and taxifolin increased ABCA1 expression in THP-1-derived macrophages. The most potent activator was silybin B (1.42-fold), whereas silybin A exhibited weak to negligible activity. Moreover, isosilybin A stimulated cholesterol efflux from macrophages in a concentration-dependent manner, possibly due to its peroxisome pro-liferator-activated receptor-gamma (PPAR)-activating properties [118]. PPARγ is a nuclear receptor that functions as a key regulator of lipid and glucose metabolism. Agonists of this receptor are used in the treatment of type 2 diabetes mellitus (DM2) as they lower blood glucose levels in DM2 patients and are also being considered for the potential treatment of other metabolic diseases, such as non-alcoholic fatty liver disease. All major silymarin flavonolignans (silybins A and B, isosilybins A and B, silychristin, and silydianin) were tested for PPARγ nuclear receptor activation in HEK-293 cells. Only isosilybin A exhibited concentration-dependent PPARγ activation (EC_50_ = 4.1 μM) [119]. The respective specific interaction of PPARγ with isosilybin A was confirmed by molecular docking. Isosilybin B did not produce a significant activation of PPARγ. Given the fact that silymarin contains ca. 8–10% isosilybin A, but only ca. 3% isosilybin B (namely in the *S. marianum* chemorace A typically used for silybin production [33]), then the above findings could well contribute to the explanation of silymarin antidiabetic activities and insulin-sensitizing properties [149] on a molecular basis.

Silychristin A (in the range of 25 μM) activated the estrogen receptor-α-dependent Nrf2-heme oxygenase-1/superoxide dismutase 2 (Nrf2-HO-1/SOD2) pathway to decrease apoptosis and upregulate glucagon-like peptide-1 (GLP-1) production in intestinal GLUTag L cells, leading to the reversal of reactive oxygen species-induced apoptosis and impairment of GLP-1 production [120]. GLP-1 is a hormone secreted mainly by intestinal L-cells; it regulates the blood glucose levels of patients suffering from DM2 via enhancing β-cell mass and potentiating glucose-dependent insulin secretion. In addition, GLP-1 can also lower postprandial blood glucose. Silychristin A thus contributes to the achievement of stable physiological glucose homeostasis. Since silychristin is one of the most important silymarin flavonolignans (about 10%, A/B 95:5), this could be another possible explanation for the antidiabetic activity of silymarin.

Streptozotocin-induced diabetes mellitus type 1 (DM1) in rats was used as a model to study the effects of silychristin A [121]. This flavonolignan lowered the glucose level, increased insulin secretion, and improved the structure of β cells. Additionally, the production of reactive oxygen species induced by streptozotocin or high glucose concentration was suppressed by silychristin A in pancreatic islet INS-1 β cells, which led to their diminished apoptosis. Silychristin A was also found to be an effective inhibitor of α-glucosidase (IC_50_ 8.2 ± 1.8 μM), which could reduce the digestion rate of carbohydrates and thus lower postprandial levels of glucose.

It should be noted here that natural silychristin is a mixture of A/B diastereomers in a ca. 95:5 ratio. Separation of the B isomer is relatively complicated [46] and silychristin B tends to co-elute with silydianin in reversed-phase columns [47]. Therefore authors claiming to work with pure silychristin A often simply work with natural silychristin containing ca. 95% of the A isomer (which is considered to be a sufficient purity level of a compound, e.g., by the standards of the Am. Chem. Soc.). This is clearly visible in the above study [121], where the HPLC profile shows the presence of a small peak(s) adjacent to the main peak of silychristin A (plausibly the 5% silychristin B). All these flavonoids can be now baseline-separated by modern gradient HPLC methods [10,11].

### 4.3. Cardiovascular Activity

The familial cardiomyopathies, hypertrophic cardiomyopathy and dilated cardiomyopathy, are relatively common, potentially life-threatening, and currently untreatable. Currently, there are no agents or interventions that can prevent or treat sarcomere cardiomyopathies. A number of flavonoids, including silymarin flavonolignans, have been shown to be able to both reduce Ca^2+^ sensitivity and/or recouple the relational relationship between troponin I phosphorylation and Ca^2+^ sensitivity, thereby restoring proper physiological functions of the myocardium [122]. Silybin B (EC_50_ 39 μM) and 2,3-dehydrosilybin B (EC_50_ 47 μM) were strong recouplers (comparable to the benchmark *epi*-gallocatechin-3-gallate (EGCG)), while silybin A and 2,3-dehydrosilybin A were ca. 10× stronger recouplers. This finding was corroborated by further experiments with thin filaments containing the phosphorylated and unphosphorylated tropomyosin E180G mutant. Interestingly, mixed silybin A/B had a lower activity than stereomer A (not the average of the A and B stereomers) indicating antagonistic activity of the A stereomer. The authors speculated that the minimal pharmacophore structure could be a flavon moiety because taxifolin (for silybin) and quercetin (for 2,3-dehydrosilybin) had comparable activities to the respective B stereomers. This, however, does not explain the differences in the activities of the respective A and B stereomers. Thus silybin B and its derivatives can provide lead compounds, being effective and specific recouplers with practical potential in the treatment of inherited cardiomyopathies.

Silymarin flavonolignans and their metabolites have biologically relevant vasodilatory properties [123]. Silybin A, silychristin, and 2,3-dehydrosilybin A demonstrated evident vasorelaxant activities, whereas 2,3-dehydrosilybin B was entirely inactive. The concentrations at which the most active compounds reached a 50% vasorelaxant effect were in the tens of μM, which can be achieved in plasma by pharmaceutical formulations of silymarin. The B stereomers were generally less potent than the A stereomers (both in silybin and 2,3-dehydrosilybin panel). The effects of the sulfated conjugates were typically comparable or even better than those of the parent compounds. In these authors, the influence of the respective flavonolignans on platelet aggregation (blood clotting) was also tested, but at physiologically achievable concentrations (<100 μM) their effects were small or negligible [123].

### 4.4. Anticancer Activity

Extensive research over the past decade has demonstrated that silymarin can inhibit proliferation of numerous tumors (e.g., lung, ovarian, breast, prostate, bladder, and co-lon); this is mediated by cell cycle arrest in G1/S phase, induction of cyclin-dependent kinase inhibitors (e.g., p15, p21, and p27), down-regulation of an apoptotic gene products (e.g., Bcl-xL or Bcl-2), inhibition of cell-surviving kinases (MAPK, AKT, and PKC), and suppression of inflammatory transcription factors (NF-kB) [150]. Silymarin can also downregulate gene products involved in tumor cell proliferation (COX -2, cyclin D1, IGF-IR, TGF-γ and EGFR), invasion factors (MMP-9), metastasis factors (adhesion molecules) and angiogenesis promoters (VEGF). Silymarin can also sensitize tumors to chemotherapeutic agents by suppressing proteins associated with multi-drug resistance (MDR) (see Section 4.9).

A landmark study demonstrating the fundamental importance of using pure compounds in cancer research was published by Davis-Searles et al. in 2005 [34]. The growth suppression by isolated silymarin flavonoids of three prostate adenocarcinoma (PA) cell lines was tested: LNCaP (CRL-1740, an androgen-dependent line from a lymph node metastasis from PA), DU145 (HTB-81, an androgen-independent line from a central nervous system metastasis from PA) and PC-3 (CRL-1435, an androgen-independent line from a bone metastasis from PA). Isosilybin B was most active in the three cell lines at 30 μM (e.g., the IC50 in DU145 is 20.5 μM for isosilybin B and 32 μM for isosilybin A); silybin B was the second most active flavonoid (in LNCaP and PC-3), whereas isosilybin A was the second most active compound in DU145 cells. Silybin A was consistently inactive in all lines tested. Isosilybin B was also most effective in repressing the topoisomerase IIa promoter: isosilybin B silybin B isosilybin A silybin A. Finally, isosilybin B was also most effective in suppressing prostate-specific antigen (PSA) secretion by LNCaP prostate carcinoma cells: isosilybin B > silybin B > isosilybin A ≈ silybin A. These results were later confirmed and corroborated by Deep et al. [125] using advanced human prostate carcinoma PC3 cells.

The molecular mechanisms of isosilybin A and B at PA were investigated by [35], who showed that these compounds provide anti-cancer activity via cell cycle arrest and apoptosis induction, which includes modulation of cyclin-dependent kinase inhibitor expression and caspase activation along with a decrease in survivin levels. Isosilybin B and A (tested concentrations 60 M and 90 M) induced apoptosis in human prostate carcinoma LNCaP and 22Rv1 cells; the effect was stronger in the 22Rv1 line, while isosilybin B was statistically more effective.

Isosilybin B (10–90 μM) suppressed androgen receptor and PSA levels in LNCaP, 22Rv1 and LAPC4 cells, but not in non-neoplastic human prostate epithelial cells PWR -1E [124]. Isosilybin B treatment inhibited the synthetic (anabolic steroid) androgen metribolone-induced nuclear localization of AR as well as PSA expression and cell growth and caused G1 arrest. Isosilybin B treatment promoted the formation of a complex between Akt, Mdm2 and AR, which promoted phosphorylation-dependent ubiquitination of AR and its degradation by the proteasome. This study clearly demonstrated at the molecular level the applicability of isosilybin B in the treatment of PA. The studies on the in vitro PA inhibitory activity of isosilybin B were further extended to in vivo experiments on a DU145 xenograft in athymic nude mice [151]. However, here an equimolar mixture of isosilybin A and B was used due to the scarcity of pure isosilybin B. Feeding isosilybin (200 mg/kg body weight/day) significantly inhibited xenograft growth after 53 days of treatment, which was equally effective or slightly more effective than si lymarin or silybin.

Interestingly, isosilybin A (90–180 μM) significantly induced apoptotic death by affecting both the extrinsic (decrease in DR5 and by cleaved caspase 8) and intrinsic pathways (activation of caspase 9 and caspase 3) of apoptosis in human prostate cancer cell lines LNCaP, 22Rv1 and LAPC4. Moreover, isosilybin A decreased the levels of phospho-Akt (serine-473) [126]. Therefore, isosilybin A activates the apoptotic system in PCA cells by targeting the Akt-NF-kB—AR pathway.

It should be noted here that most effects occurred in vitro at concentrations of 30 M, which is not easily achieved in vivo (practically, a concentration of 10 M can be reached in plasma). However, the promising potential of isosilybin B for treatment PA is limited by its low content of silymarin (about 4%) and by its relatively complicated isolation on a large scale [55]. Nevertheless, thanks to its low toxicity, isosilybin B is an interesting lead compound for prostate cancer suppression or treatment. Some clever marketers of silymarin food supplements place the “content of isosilybin B complex” on the information label as a form of promotion (e.g., Bexoliv by Bionova Lifesciences, IN and many others). This, however, does not mean that the isosilybin B content in silymarin in these products was increased above the standard level.

**Figure 11:**
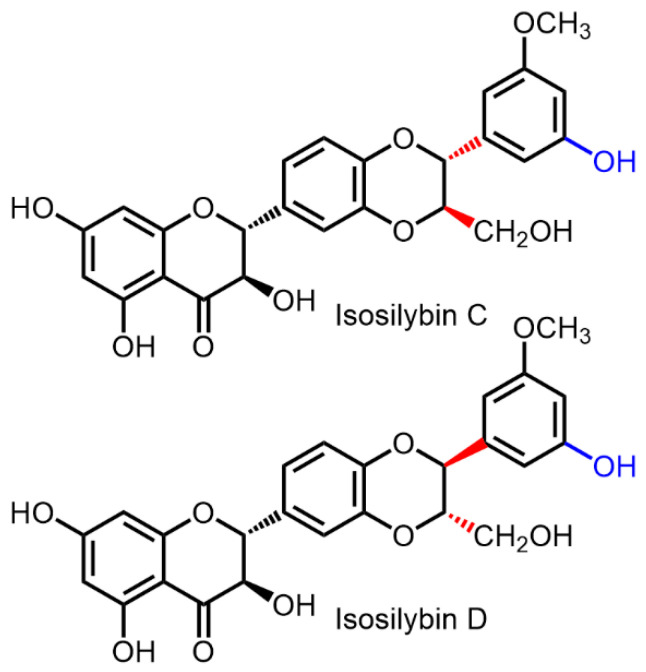
Structures of minor silymarin components isosilybin C and isosilybin D [53].

Sy-Cordero et al. [53] performed large-scale isolation of all major silymarin flavonolignans and managed to isolate also two new flavonolignan minorities, e.g., isosilybin C and isosilybin D (Figure 11). They tested all known and two new flavonoids for cytotoxic/antiproliferative activity against three prostate cancer cell lines DU145, PC-3, and LNCaP. Both isosilybin C and isosilybin D had lower activity compared to silybins A and B and isosilybins A and B—IC_50_ of isosilybins C and D in all cell lines were ca twice higher than that of the best compound—isosilybin B, which indicates that an *ortho*- position of substituents at E-ring is more favorable for anticancer activity than the *meta*- position.

Anti-tumor efficacy has been demonstrated in vivo for silybin A, silybin B, isosilybin A, and isosilybin B on the DU145 human prostate xenograft in athymic nude mice; they can inhibit tumor growth after oral treatment with doses of 50 and 100 mg/kg body weight [127]—isosilybin B was most effective. The flavonolignans tested inhibit tumor angiogenesis biomarkers (nestin and CD31) and signaling angiogenesis molecules (VEGF, VEGFR1, VEGFR2, phospho-Akt, and HIF-1a) without affecting angiogenesis in normal healthy tissue. Further testing with human umbilical vein endothelial cells (HUVEC) showed that these diastereoisomers target the cell cycle, apoptosis, and the VEGF-induced signaling cascade. This work demonstrated the anti-angiogenic activity of optically pure flavonolignans and suggests their applicability in prostate cancer an-gio-prevention.

**Figure 12:**
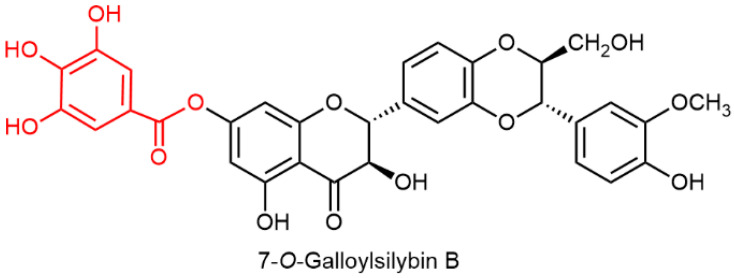
Structure of 7-*O*-galollylsilybin B, a potent antiangiogenic compound [99].

The antiangiogenic activity of silybin A and B and some galloylated silybin derivatives was demonstrated before the above study in 2011 [99]. Natural mixed silybin showed relatively poor antiangiogenic activities, while its B stereoisomer was more active than silybin A. A series of *O*-galloylated silybin derivatives were prepared (substitution at C-3, C-7 and C-20 position) based on structural analogy with EGCG, which is one of the most active antiangiogenic components found in green tea [152]. Silybin B was the most effective in the HUVEC proliferation assay (IC_50_ 6.5 μM, compared with 8.8 μM for silybin A). The most effective galloylated derivative was 7-*O*-galollylsilybin B (IC_50_ 4.3 μM compared with 6.1 μM for its A derivative) (Figure 12). Additionally, 7-*O*-galollylsilybin B was most potent as a HUVEC growth inhibitor (IC_50_ 7.9 μM, compared with 60.5 μM for silybin B). Strong anticancer effectivity of this derivative was later confirmed in the inhibition of human bladder cancer HTB9 cells [86].

Silybin A and silybin B induced cell apoptosis of the human chronic myeloid leukemia K562 cells with a similar potency via the mitochondrial and MAPK pathway. Mixed silybin displayed weaker cancerostatic effects than each pure diastereomer 132.

Serine/threonine-protein kinase B-Raf (BRAF kinase)—the product of proto-oncogene B-Raf and SMO protein (G protein-coupled receptor—a component of the hedgehog signaling pathway) are two major targets in current anticancer therapy. In silico docking studies demonstrated that both silybins A and B and dehydrosilybins A and B have very similar docking scores to the binding sites of BRAF and SMO kinases similarly to vemurafenib and vismodegib that are established inhibitors of the proteins used in cancer treatment [136]. This finding was confirmed by in vitro studies with BRAF kinase where dehydrosilybin B showed the best inhibitory activity (IC_50_ = 25 µM), ca. three times better than its A stereomer and both silybins A and B. Strong dose-dependent binding of both dehydrosilybin A and B was also demonstrated in the BODIPY-cyclopamine SMO binding assay. This combined in silico/in vitro approach is a nice demonstration of the molecular effect of 2,3-dehydrosilybin B. The cytotoxicity experiments further confirm the cytotoxic properties of the compounds in malignant skin cell lines by showing higher activity in A-375 cells than in A-431 and HaCaT [136].

In addition to natural flavonolignans, their derivatives, including those stemming from optically active parent compounds, were also tested for the inhibition of cancer cell lines. A series of silybin B methyl derivatives were evaluated for their antiproliferative effect against human prostate cancer lines DU-145, PC-3, and LNCaP cells, and also human hepatoma line Huh7.5.1. All the methyl derivatives were equal to or more potent than the parent compound, with 7,20-*O*-dimethylsilybin B being the most potent derivative (ca 6 × more potent) [97]. It was found that 7-*O*-Methylation of all major flavonolignans of silymarin significantly increased their cytotoxicity in a human Huh7.5.1 hepatoma cell line, with the average fold change being approximately 6, lowering the IC_50_ from the average range of ~70 μM in the parent flavonoids to ~10–15 μM in the respective 7-*O*-methyl derivatives [96].

Vue et al. [153] prepared a large series of 7-*O*-alkylsilybin and 7-*O*-alkyl-2,3-dehydroslilybins, however only from the racemic flavonoids. They concluded that 7-*O*-methyl and 7-*O*-ethyl silybins were the most potent compounds with potency against the LNCaP cell line that was enhanced by ca. two orders of magnitude. The same types of derivatizations of 2,3-dehydrosilybin were identified as the ideal, having the highest potency towards both androgen-dependent LNCaP and androgen-independent PC-3 prostate cancer cell lines.

7-*O*-Methyl- and 7-*O*-galloyl silybins A and B, parent compounds and 2,3-dehydrosilybins A and B were tested for the inhibition of human bladder cancer cells HTB9, colon cancer HCT116 cells, and androgen-insensitive prostate cancer cells PC3 [86]. The introduction of a methyl or galloyl group at C-7 in silybin significantly improves the inhibitory effect on prostate cancer cells, and no appreciable difference in the in vitro inhibitory effect was observed between two optically pure derivatives, even between the derivative present as a mixture of diastereomers and the, respectively, enantiomerically pure derivative. However, a significant difference between two diastereomers was observed in the inhibition of HTB9 cells (human bladder carcinoma); the strongest difference was observed with 7-*O*-galloylsilybin B, which was about 2× more efficient than its A diastereomer (in the concentration range of 5–10 μM). In a colony inhibition test with HTB9 cells, the most efficient (concentration 20 μM) was 2,3-dehydrosilybin B followed by 2,3-dehydrosilybin A; silybins A and B and the respective 7-*O*-methyl silybins were ca. half as effective. These results demonstrate that the anti-cancer efficacy of silybin could be significantly enhanced by substitution mostly at the C-7 position (methylation, galoylation) and 2,3-desaturation. Furthermore, these results were consistent with three lead silybin derivatives in three completely different human cancer cell lines, therefore the observed anti-cancer activity is not cell-line specific.

All pure silymarin flavonolignans were tested as potential antitumor-promoting agents by short-term 12-*O*-tetradecanoylphorbol-13-acetate-induced Epstein-Barr virus early antigen activation assay in Raji cells (derived from the B-lymphocytes of Burkitt’s lymphoma). They all demonstrated good inhibitory activity; silychristin B and A were the most active compounds [128].

A very detailed and comprehensive review on the effects of all the flavonolignans tested for prostate cancer management was published by Vue and Chen [154]. This commendable material compares in detail all the flavonolignans tested in different PA models, reviews the active anticancer concentrations, and provides a deep and systematic overview of the promise and potential of flavonolignans in the treatment of prostate cancer.

### 4.5. Inhibition of Hepatitis C Virus; Antiviral Activity

There exist a plethora of papers describing numerous antiviral activities of various silymarin or crude silybin preparations, reviewed by Liu et al. [155]. The most frequent target for silymarin intervention is hepatitis C virus (HCV) [156] but also dengue virus, influenza A virus, human immunodeficiency virus, togaviruses—chikungunya virus and mayaro virus, and hepatitis B virus. There is also a report suggesting COVID-19 treatment with silybin [157], based on the fact that silybin (mixed) can inhibit signal transducer and activator of transcription Stat3 [150], and Srivastava et al. [158] identified silybin B (among others) as a potential anti-COVID drug based on its in silico docking to the main protease and to the spike protein crystal structures (this activity is rather speculative, as no wet experiments confirmed these findings). Clinical trials with silymarin were accomplished in patients with chronic hepatitis C. Even though most of these studies demonstrated very high effectiveness of silymarin, there are only a handful of studies dealing with pure isolated silymarin flavonolignans [155].

A pioneering study that investigated isolated silymarin flavonolignans in hepatitis C-infected Huh7 human hepatoma cells in detail was accomplished in a coordinated study of two prolific groups from Seattle and Greensboro [130]. Isosilybin A, taxifolin, and silybin (IC_50_ 30–80 μM) were the most effective hepatoprotectants, as they demonstrated potent activity in four of the six accomplished assays (antiviral effects based on HCV protein expression; inhibition of HCV RNA replication; inhibition of HCV NS5B polymerase; inhibition of HCVcc-induced oxidative stress; inhibition of TNF-α-induced NF-κB transcription; and inhibition of the TCR-mediated induction of T-cell proliferation), followed by silybin A and silybin B, which were active in three of the five assays. Isosilybin B was toxic to the Huh 7 cells above 10 μM, therefore its antiviral activity in this model can be ascribed to its general cytotoxicity. All the measured activities, e.g., antiviral, antioxidant, anti-inflammatory, and immunomodulatory, are all likely to be directly related to its well-described hepatoprotective actions.

Ahmed-Belkacem et al. [131] investigated all the major silymarin flavonolignans silybin A and B, isosilybin A and B, silychristin, and silydianin prepared in the form of water-soluble bishemisuccinate salts (Legalon SIL—commercial intravenous preparation of silybin prepared by the Madaus Co., Cologne, Germany). Silybin A and B inhibited JFH1 replication in cell culture, with an EC_50_ in the range of 20–40 µM. Silybin A and B and isosilybin A and B were potent inhibitors of HCV genotype 1b replicon replication in cell culture, with an EC_50_ on the order of 1 µM. Silychristin and silydianin did not affect HCV RdRp, nor on HCV replication, and they are therefore ineffective in HCV treatment. Thus, in conclusion, silybin A and B, as well as Legalon SIL inhibit HCV replicon and JFH1 replication in cell culture.

The IC_50_ value for inhibition of HCV infection by silybin B is about 40 to 80 µM [130]. Methylation of silybin B significantly increased the antiviral activity of the respective derivatives. The silybin B derivatives tested inhibited HCV infection at lower concentrations, with the 7,20-*O*-dimethyl silybin B (at concentrations of 2–10 µM) being the most active. These data show that the antiviral activity of the methylated silybin B analogs was in the following order: 7,20-*O*-dimethyl- > 7-*O*-methyl- > 5,7,20,23-*O*-tetramethyl- > 5,7,20-*O*-trimethyl- > silybin B [97].

There are two hypotheses for the anti-HCV activity of silymarin components: (i) the compounds could induce cellular antiviral effectors [130] and (ii) they could directly inhibit vital HCV functions [131]. Neither of these hypotheses has been excluded so far.

### 4.6. Antiparasitic Activity

The antiparasitic activity of silymarin flavonolignans and some other flavonoids was recently reviewed by Faixová et al. [159], demonstrating that some flavonoids and polyphenols can also be successfully used in parasitology—particularly on medically important flatworms such as *Raillietina* spp., *Fasciola* spp., *Leishmania* spp., *Schistosoma* spp., *Echinococcus* spp., and on the (model) cestode *Mesocestoides vogae*. Most of the existing papers worked with mixed preparations or with silymarin. There are, however, recent papers that also describe in detail the application of pure flavonolignans in parasitology [132].

Antiproliferative activities of silymarin and its pure components silybin A and B, isosilybin A, silychristin A, silydianin, 2,3-dehydrosilybin A and B, 2,3-dehydrosilychristin A, 2,3-dehydroisosilybin A, and 2,3-dehydroisosilydianin were tested for the inhibition of *Leishmania infantum (L. chagasi)* and *L. donovani* promastigotes (causative agents of human and canine leishmaniase) [132]. In the range of concentrations ca. 1–120 μM, the tested flavonolignans exhibited substantial differences in their antileishmanial activity. The antileishmanial effects against *L. infantum* were modest and less than 50% of the growth of the control cultures: dehydrosilydianin > dehydroisosilybin A > dehydrosilychristin A > silychristin A > dehydrosilybin B. However, dehydroisosilybin A exhibited the highest inhibitory activity against *L. donovani* promastigotes (88%), while the activity of the other flavonolignans below was below 50% of the control. Dehydroisosilybin A showed a synergistic effect with amphotericin B against *L. infantum*. The maximal activity against amastigotes of *L. infantum* was observed for 10 μM dehydrosilybin A and B stereoisomers. Amastigotes are the actual parasite stage that causes the disease, thus these results justify the use of amastigotes for further characterization of the potential antileishmanial effects of flavonolignans. Thanks to the generally low toxicity of all flavonolignans used, they may be promising for complementary antileishmanial therapy.

### 4.7. Neurological Activity

A seminal paper pointing to the potential of silymarin flavonoids and the importance of their stereochemistry in Alzheimer’s disease was published by Filippopoulou et al. [134]. The effects of 2,3-dehydrosilybin A and B on the progression of Alzheimer’s disease (AD) were tested in nematode *Caenorhabditis elegans* AD models. A paralysis assay in the GMC101 transgenic strain expressing human amyloid β1-42 in body wall muscle cells followed by self-assembly of the amyloid peptide into neurotoxic aggregates, causing nematode muscle paralysis, showed a significantly delayed rate of paralysis after treatment with 2,3-dehydrosilybin A and B, accompanied by reduced levels of both total and oligomeric amyloid β (Aβ) species. This finding was confirmed with human SH-SY5Y neuroblastoma cells exposed to the Aβ overproducing cell line 7PA2. Analysis revealed reduced levels of total and oligomeric Aβ species after 2,3-dehydrosilybin A and B treatment. Thus, 2,3-dehydrosilybin A and B were protective against oxidative stressors and Aβ proteotoxicity in nematodes and human cells. In addition, a significant dose-dependent lifespan extension of nematode *C. elegans* was also observed with both 2,3-dehydrosilybin A and B (approximately equal activity of both enantiomers) in a concentration range of 10–50 μM. The extension of lifespan is presumably caused by the inhibition of FGT-1 (facilitative glucose transporter isoform 1) in *C. elegans* by 2,3-dehydrosilybin A and B.

The abovementioned findings were later confirmed and extended by Sciacca et al. [101], who tested all four stereomers of silybin A and B and 2,3-dehydrosilybin A and B for the inhibition of Aβ aggregation and proteotoxicity in the transgenic *C. elegans* strain CL4176 expressing human Aβ. This study demonstrated that silybin B (ca 10× more effective than silybin A and ca. 5× more effective than dehydrosilybins) is the most effective at counteracting Aβ proteotoxicity. This finding again clearly demonstrates the central role of stereochemistry in determining the neuroprotective potential of silybins.

Amyloidosis also occurs in other proteins, and may also cause disorders in addition to neurological ones. Lysozyme is extensively distributed in a variety of tissues and body fluids, and its fibrillation (often caused by mutations) causes numerous debilitating pathologies. Chen et al. [133] demonstrated that the guanidine-induced aggregation process of hen egg-white lysozyme (model) can be effectively inhibited by silybin B, while silybin A has considerably lower activity. Results were supported by the molecular docking of both silybins, which exhibited binding energy of −7.1 kcal/mol and kI of 6.28 μM for silybin A and −8.69 kcal/mol and inhibition constant kI of 0.426 μM for silybin B. The difference between silybin diastereomers in binding to HEWL was mainly caused by the C-20 and C-23 hydroxyls [133]. Silybin B also increases the viability of human SH-SY5Y neuroblastoma cells exposed to HEWL fibrils; the effective concentrations of both silybins were in the range of 224–448 μM, which is, however, beyond the range accessible in vivo.

Recent studies have shown that conjugation of a trehalose moiety to silybin A and B (Figure 8) increases water solubility without significantly affecting anti-aggregation properties [102]. An NMR study showed that silybins may act by shielding toxic Aβ40 surfaces formed by *N*-terminal and CHC-Aβ regions, and that the aromatic ring A of silybin is the primary site for interaction with Aβ-oligomers.

However, the question remains of whether silybin (or its derivatives) can cross the blood-brain barrier and be effective neuroactive drugs.

### 4.8. Inhibition of Drug-Metabolizing Enzymes

Numerous aromatic compounds, and often flavonoids, are typical inhibitors of CYPs and other biotransformation enzymes—a typical example being furanocoumarins from grapefruits. Inspired by previous observations with silymarin inhibition of the biotransformation of some drugs, Brantley et al. [143] investigated isolated flavonolignans—silybins A and B and isosilybins A and B. It was clearly shown that particular flavonolignans inhibit the metabolic activity of CYP 2C9 with different intensities. Each flavonolignan inhibited the 7-hydroxylation of (*S*)-warfarin in a concentration-dependent manner in the order silybin B > silybin A >> isosilybin B > isosilybin A. The strongest inhibitor silybin B lowered warfarin 7-hydroxylation activity with IC_50_ = 6.7 μM for CYP 2C9*1 and IC_50_ = 8.2 μM for human liver microsomes. The IC_50_ values of the second strongest inhibitor silybin B were ca. twice that for silybin B. This finding warns against the use of larger doses of silybin preparations in combination with the administration of warfarin as an anticoagulant treatment, and possibly other drugs metabolized by CYP 2C9. Warfarin has a narrow therapeutic window, and therefore its accumulation in the body due to its slower metabolism may cause uncontrolled bleeding. Micromolar concentrations of silybin can be achieved in vivo, although most of it remains in conjugated forms whose inhibitory activities are not yet known.

Enterocytes, analogously to hepatocytes, express a large number of biotransformation enzymes, thus influencing the extent of first-pass xenobiotic metabolism. The inhibition of CYP 3A by eight isolated silymarin constituents was investigated in human liver microsomes and intestinal microsomes with midazolam (a benzodiazepine derivative used for anesthesia and sedation) as a substrate [144]. Silybin A and silybin B were identified as the strongest inhibitors (IC_50_ 23 μM and 27 μM, respectively) specifically in the intestinal microsomes, where the concentrations of the respective flavonoids can reach up to 100 μM, particularly with gram doses of silybin [160].

In addition to CYPs being the typical enzymes of biotransformation phase I, phase II biotransformation enzymes in enterocytes are also influenced by silymarin flavonolignans. The inhibitory activity of seven silymarin constituents towards UDP-glucuronosyl transferase 1A (UGT 1A), UGT 1A8, and UGT 1A10, with 4-methylumbelliferone as the glucuronyl acceptor, was investigated by Gufford et al. [81]. The strongest inhibitors were both silybin A and B (their activities were the same within the range of experimental error) with IC_50_ ca. 28 μM for UGT 1A, 5.8 μM for UGT 1A8, and 2.7 μM for UGT 1A10. The authors also monitored the respective inhibitor depletion, since they were also substrates for the respective UGTs in the intestinal microsomes. Although 4-methylumbelliferone is just a model substrate, the inhibitory activity of the silymarin flavonolignans on biotransformation systems must be carefully evaluated in terms of potential drug-drug interactions.

A direct systemic effect of the above-mentioned inhibitory activities of silymarin constituents, especially on the UGT 1A, is the increase in systemic and hepatic bilirubin concentrations observed after the administration of milk thistle preparations in mice [146]. Slightly elevated plasmatic bilirubin concentration has been shown to be protective against some oxidative stress-related diseases, including cardiovascular disease, cancer, and autoimmune or neurodegenerative diseases. This beneficial effect is observed in inherited benign hyperbilirubinemia, known as Gilbert syndrome [161]. Screening of the entire panel of tested compounds in the HepG2 cell line revealed significant underexpression of UGT 1A1 mRNA with silybin and 2,3-dehydrosilybins A and B. These results were reflected by inhibition of UGT 1A1 activity as determined for 2,3-dehydrosilybins A and B (IC_50_ 2 μM and 4 μM, respectively) and an increase in intracellular concentrations of bilirubin (approximately 800% and 700% of control, respectively). The strongest inhibitors were consecutively tested in the in vivo experiments in mice. After the *i.p.* application of 2,3-dehydrosilybins A and B (50 mg/kg), there was a significant downregulation of liver UGT1A1 mRNA (ca 46% of the control). In both the *p.o.* and *i.p.* administration of 2,3-dehydrosilybins A and B, a significant elevation of serum bilirubin concentrations (125%—*p.o.* and 160%—*i.p.* of the controls) and also a decrease in lipoperoxidation in the liver was observed with both 2,3-dehydrosilybins [146]. The respective effects of both 2,3-dehydrosilybin enantiomers A and B were comparable within the range of experimental error. Unconjugated hyperbilirubinemia was observed as an adverse effect (maybe positive) in prostate cancer patients treated with high doses of silybin [160]. In natural silmarin, 2,3-dehydrosilybin is present, in variable amounts up to 5%. Thus, this phenomenon may contribute to the hepatoprotective effects of silymarin observed in many, although not all, clinical studies.

### 4.9. Multidrug Resistance Activity

Multidrug resistance (MDR) is a major challenge in both cancer chemotherapy and antibiotic treatment of bacterial infections. Compounds with inhibitory activity towards these proteins are promising for adjunctive therapy of such pathologies. Several fla-vonoids have been found to reverse both bacterial multidrug and antineoplastic resistance by inhibiting some drug transporters [162].

Very recently, a series of reports appeared that describe the activity of various silymarin flavonolignans in modulating multidrug resistance.

Silychristin A (one of the most abundant flavonolignans in silymarin) and its derivatives have a broader range of biological activities than previously supposed. In addition to their potent anti-inflammatory and antioxidant (decrease in NO production) activities demonstrated in vivo, both silychristin A and its derivatives are able to inhibit P-glycoprotein (P-gp) in a concentration-dependent manner, thereby sensitizing the multidrug-resistant ovarian cancer cell line (HOC/ADR, A2780/ADR) to the cytotoxic effects of doxorubicin [141]. Silychristin derivatives inhibit P-glycoprotein (transmembrane ef-flux pump) in a concentration-dependent manner in the order 2,3-dehydrosilychristin A (IC50 15.6 μM) ≥ anhydrosilychristin > silychristin A > isosilychristin. Additionally, 2,3-dehydrosilychristin A and silychristin A (20 μM) sensitized doxorubicin-resistant human ovarian cancer cells (HOC/ADR) approximately 5× to doxorubicin. Anhydrosilychristin decreased the expression of the P-gp (ABCB1) and ABCG2 genes by 28% and 40%, respectively. These genes are typically overexpressed up to 400-fold in the doxorubicinresistant cell line (the sensitive cell line expresses these genes at negligible levels). Thus, the mechanism of action of silychristin A and 2,3-dehydrosilychristin A is direct inhibition of the transporter, in contrast to anhydro- and isosilychristin, which modulate the MDR phenotype by inhibiting P-gp expression.

In addition to silychristin A, silybin B also acted directly on P-gp in vitro and down-regulated the expression of the corresponding MDR genes, while altering the expression of P-glycoprotein (P-gp, ABCB1), multidrug resistance-associated protein 1 (MRP1, ABCC1) and breast cancer resistance protein (BCRP, ABCG2) [140]. Nevertheless, silybin B was approximately 3× worse than silychristin A in terms of doxorubicin sensitization rate of doxorubicin-resistant human ovarian cancer cells.

Silychristin (in reality, approximately 95% silychristin A) has been identified as the most potent inhibitory component of silymarin (IC_50_ = 0.11 μM) of monocarboxylate transporter 8 (MCT8), which is a highly specific thyroid hormone transmembrane transporter (THTT) that enables the import and export of thyroid hormones in cells [139]. Silychristin also inhibited estradiol-17β-glucuronide uptake by some organic anion transporters (OATP1B1, OATP1B3, and OATP2B1) with an IC_50_ in the M range [137], which are also known to act as THTTs. Silybin (mixed) was a much weaker MCT8 inhibitor [139]. It is known that inhibition of THTT can delay cancer onset or reduce its belligerence. On the other hand, the safety of silymarin (containing up to 10% silychristin) needs to be carefully evaluated and the administration of high doses over a prolonged period of time carefully weighed in relation to possible dysregulations of the thyroid hormone axis.

Flavonolignans were identified to be a novel class of Na^+^/K^+^-ATPase (from porcine kidney) inhibitors, and particularly 2,3-dehydrosilychristin A and 2,3-dehydrosilydianin (IC_50_ 38 μM and 36 μM, respectively) were rather strong inhibitors; silychristin was a ca. 3× weaker inhibitor than its dehydroderivative and silybin, silydianin, and 2,3-dehydrosilybin had no or negligible inhibitory activity. The flavonolignans differ from cardiac glycosides currently used in the treatment of Na^+^/K^+^-ATPase disorders because their binding sites are different, as demonstrated by in silico docking [138].

### 4.10. Other Biological Activities

Silymarin flavonolignans have been identified as quite potent inhibitors of protein tyrosine phosphatase 1B (PTP1B), T-cell protein tyrosine phosphatase, and vaccinia H1-related phosphatase [39]. Inhibition of PTP1B increases insulin sensitivity, improves glucose tolerance and resistance to fat-induced weight gain, however without adverse effects. PTP1B inhibitors also play a positive role in controlling breast and colorectal cancer [39]. The best (non-competitive) inhibitor of PTP1B was iso-silybin B (K_i_ 1.03 μM), followed by silybin A (K_i_ 1.25 μM), isosilybin A (K_i_ 2.25 μM), Silychristin A (K_i_ 3.95 μM) and silybin B (K_i_ 4.05 μM); silydianin and dehydrosilydianin showed no inhibitory activity. Other cytosolic protein tyrosine phosphatases were tested against the best PTP1B inhibitors to demonstrate the selectivity of their PTP1B inhibition. Of all the compounds tested, silybin A showed the best inhibitory selectivity for PTP1B over other tyrosine phosphatases.

Tyrosinase is a monooxygenase responsible for (in vivo) melanin production. Its inhibitors have potential applications in cosmetics and medicine. Silymarin flavonolignans were identified to be potent tyrosinase (from mushroom) inhibitors with IC_50_ = 1.7–7.6 μM (substrate l-tyrosine) and IC_50_ = 12.1–44.9 μM (substrate l-3,4-dihydroxyphenylalanine (l-DOPA)). The most efficient was silybin AB (unfortunately authors failed to test separated silybin diastereomers), followed by isosilybin A and silydianin, all with a mixed type of inhibition. The tyrosinase activity of the tested flavonolignans is rather potent compared to the positive control—kojic acid—with IC50 15.3/37.1 μM (l-Tyr/l-DOPA) [142].

Silybin A lowered nitrosative stress in both a PC12 cell line (from the pheochromocytoma of the rat adrenal medulla) and human hepatoblastoma HepG2 cells. Low concentrations of silybin A (in the range of 25–100 μM) improved basal mitochondrial membrane potential and ATP levels in HepG2 cells, but less so in PC12 cells [135]. Silybin A may be helpful in neurological disorders, although it did not generally improve mitochondrial function. Neither silymarin flavonolignans nor silybin B were studied in this work.

## 5. Conclusions and Perspectives

This review surveyed in detail specific optically pure flavonolignans from silymarin (milk thistle crude extract) in terms of their chemical behavior and mainly in terms of their biological interactions. Silymarin has been used for millennia to treat various problems, mostly gastrointestinal. It is considered generally safe with a minimum of quite negligible adverse effects. The use of this phytotherapeutic is sometimes accompanied by controversy and often leads to non-standard or non-reproducible results. As shown above, the isolated constituents of silymarin have distinctly different properties and effects. The inherent complexity and non-standardized composition of silymarin is the main reason for these controversies. The relative composition of silymarin constituents varies significantly between and among preparations. Now, it is clear that silymarin flavonolignans do not act as antioxidants in vivo (they are very poor antioxidants even in vitro), but they act as specific ligands of certain biological targets, as in the “lock-and-key” concept. This highlights the utmost importance of the use of optically pure compounds as profound differences between the respective diastereomeric pairs of the respective components have been unequivocally demonstrated.

It should be borne in mind that most effects occurred in vitro at concentrations of 30 μM, which is not easily achieved in vivo; in plasma, a concentration of 10 μM (silybin A and B) can be practically achieved.

The future of “silymarin application” lies not in testing this extract for more and more applications, often in vain, but in the use of individual components that can be applied directly or used as valuable lead structures, and in the study of truly molecular effects. With the preparative and scalable separation methods available today, pills made from isosilybin B in the treatment of prostate cancer, silychristin A and dehydrosilychristin in adjunctive antibacterial or cytostatic therapy or in diabetes treatment, dehydrosilybin B in the therapy of cardiomyopathies, and many others are conceivable. A great potential of the somewhat neglected silychristin has only recently been discovered and is now awaiting practical application. A major advantage of these pure silymarin constituents—thanks to their proven safety—is their potential for long-term use often as adjunctive and supportive drugs, based on solid knowledge of their detailed molecular interactions.

## Figures and Tables

**Table 1:** Biological effects of optically pure silymarin flavonolignans and derivatives.

Biological Effect	MODEL USED	Active Substance	Reference
Estrogen receptor activation	ER- and AhR-mediated luciferase activity in cell lines T47D.Luc and H4IIE.Gud.Luc	Silybin B	[117]
PPARγ activation	PPARγ luciferase reporter transactivation nuclear receptor activation in HEK-293 cells	Isosilybin A	[118]
Cholesterol efflux transporter ABCA1 expression induction	ABCA1 protein expression in THP-1 macrophages	**Silybin B ^a^** Isosilybin A Silychristin Isosilychristin	[119]
(Nrf2-HO-1/SOD2 pathway activation; Decrease in apoptosis and upregulation of GLP-1	Intestinal murine enteroendocrine L-cell line GLUTag apoptotic activity and production of GLP-1	Silychristin A	[120]
α-Glucosidase inhibition; improvement of oral sucrose tolerance	α-Glucosidase from *Saccharomyces cerevisiae*	Silychristin A	[121]
Lowering fasting blood glucose and stimulation of insulin secretion by improving the viability of pancreatic β cells	Streptozotocin-induced *diabetes mellitus* type 1 in rats	Silychristin A	[121]
Inhibition of respective phosphatases	Protein tyrosine phosphatase 1B (human recombinant); T-cell protein tyrosine phosphatase (human recombinant); Vaccinia H1-related phosphatase (human recombinant)	**Silybin A** Silybin B Isosilybin A **Isosilybin B** Silychristin A Silychristin B Isosilychristin A Dehydrosilychristin A Silydianin	[39]
Ability to re-couple troponin in thin myofilaments; Restoration of inherited cardiomyopathy	Difference between motility in phosphorylated and unphosphorylated forms of thin filaments reconstituted from rabbit skeletal or mouse cardiac muscle α-actin with TPM1 E180G HCM mutation.	**Silybin B ^a^** Silybin A **2,3-Dehydrosilybin B** 2,3-Dehydrosilybin A Silychristin Silydianin	[122]
Vasodilatory activity	Rat aortic ring contraction/dilatation	**Silybin A ^a^** Silybin B 2,3-Dehydrosilybin A 2,3-Dehydrosilybin B 2,3-Dehydrosilychristin Respective sulfated metabolites	[123]
Antiproliferative activities against LNCaP, DU145, and PC3 human prostate carcinoma cell lines; Suppresion of DNA topoisomerase IIa gene promoter in DU145 cells	LNCaP (CRL-1740, androgen-dependent line from lymph node metastasis of PA), DU145 (HTB-81, androgen-independent line derived from central nervous system metastasis of PA), and PC-3 (CRL-1435, androgen-independent line derived from bone metastasis of PA); topoisomerase IIA promoter-reporter transfection and reporter activity assay in DU145 cells.	Silybin A Silybin B Isosilybin A **Isosilybin B ^a^**	[34]
Growth inhibition and cell death and significant G1 arrest and apoptosis; Decrease in the levels of both cyclins (D1, D3, E, and A) and Cdk2, Cdk4, and cell division cycle 25A, increase in p21, p27, and p53 concentrations	Human prostate carcinoma cell lines LNCaP and 22Rv1.	Isosilybin A **Isosilybin B ^a^**	[35]
Decrease in AR and PSA; Increase in phosphorylation of Akt (Ser-473 and Thr-308) and Mdm2 (Ser-166)	LNCaP cells with single AR mutation (T877A), LAPC4 cells with wild-type AR, and androgen-independent 22Rv1 cells with mutated AR (H874Y)	Isosilybin B	[124]
Inhibition of colony formation of cell lines tested; Induction of cell cycle arrest (in PC3 line)	HPC PC3 and LNCaP cells	Silybin A Silybin B Isosilybin A **Isosilybin B ^a^**	[125]
Activation of apoptotic pathways in PA cells via targeting the Akt-NF-kB-AR pathway	HPC 22Rv1, LNCaP, and LAPC4 cells	Isosilybin A	[126]
Competitive anti-angiogenetic effects;Down-regulation of prostate tumor angiogenesis biomarkers—CD31 and nestin; Target VEGF-induced signaling cascade; Down-regulation of the cyclin A, D1, D3, and E levels; Down-regulation of the level of Cdk4 and Cdk6, but not of Cdk2	Athymic (*nu/nu*) nude mice with DU145 cell line xenograft	Silybin A Silybin B Isosilybin A **Isosilybin B**	[127]
Antiproliferative/cytotoxic activity	HPC DU-145, PC3, and LNCaP cells	Silybin A Silybin B Isosilybin A **Isosilybin B** Isosilybin C Isosilybin D	[53]
Cell line growth inhibition in 72 h assay	HPC DU-145, PC-3, and LNCaP cells and a HH cell line Huh7.5.1	Silybin B Respective mono-, di-, tri-, and tetra-*O*-methylated derivatives	[97]
Cell line growth inhibition	HH Huh7.5.1 cell line	Silybin A Silybin B Isosilybin A Isosilybin B Silychristin Silydianin All respective 7-*O*-methyl derivatives	[96]
Inhibition of Epstein-Barr virus early antigen activation	12-*O*-Tetradecanoylphorbol-13-acetate-induced Epstein-Barr virus early antigen activation in Raji cells	Silybin A Silybin B Isosilybin A Isosilybin B Silychristin A **Silychristin B** Silydianin	[128]
Inhibition of cell growth and colony formation of tested cell lines—trypan blue dye exclusion assay	Human bladder cancer HTB9, colon cancer HCT116, and HPC PC3 cells	Silybin A Silybin B 2,3-Dehydrosilybin A 2,3-Dehydrosilybin B 7-*O*-Methylsilybin A 7-*O*-Methylsilybin B 7-*O*-Galloylsilybin A 7-*O*-Galloylsilybin B	[86]
Cell growth inhibition and apoptosis induction	Chronic myeloid leukemia K562 cells	Silybin A Silybin B	[129]
HUVEC viability inhibition; Inhibition of HUVEC proliferation; Inhibition of endothelial cell differentiation (tube formation) and migration	HUVEC	Silybin A Silybin B 7-*O*-Galloylsilybin A **7-*O*-Galloylsilybin B**	[99]
Inhibition of HCVcc infection, NS5B polymerase activity, HCVcc induced oxidative stress, TNF-α-induced NF-κBtranscription, and TCR-mediated induction of T-cell proliferation	HCV infected Huh7.5.1 HH cells by virus strain JFH-1	Silybin A Silybin B Isosilybin A Isosilybin B Silychristin Isosilychristin Silydianin Taxifolin	[130]
HCV RNA-dependent RNA polymerase inhibition and NS3/4A protease inhibition; Inhibition of JFH1 replication in Huh7 cells	Recombinant HCV RdRp (NS5BΔ21) RNA-dependent RNA polymerase, HCV NS3/4A protease, and HCV JFH1 infected Huh7 cells	Silybin A Silybin B Isosilybin A Isosilybin B Silychristin Silydianin Plus all the above flavonolignans in the water-soluble form of bishemisuccinate di-Na salts	[131]
Inhibition of multiplication of promastigotes in vitro and ex vivo on intracellular amastigotes of *Leishmania infantum (L. chagasi)* and *L. donovani*	Intracellular amastigotes of *L. infantum* and *L. donovani*, causative agents of human and canine visceral leishmaniasis. *L. infantum* Li UCM9 (M/CAN/ES/2001/UCM9) and Li BCN150 (M/CAN/ES/96/BCN150 zymodeme MON-1) and *L. donovani* (MHOM/SD/43/124).	Silybin A Silybin B Isosilybin A Silychristin A Silydianin 2,3-Dehydrosilybin A 2,3-Dehydrosilybin B 2,3-Dehydrosilychristin A **2,3-Dehydroisosilybin A** 2,3-Dehydrosilydianin	[132]
Lifespan extension of *C. elegans*; Inhibition of FGT-1 in *C. elegans*; antiaggregation activity ofAβ in *C. elegans* GMC101 strain overproducing Aβ and protection against toxicity of Aβ in human neuroblastoma SH-SY5Y cells	*Caenorhabditis elegans* N2, DR26, CL2179, CL2331, and GMC101 strains; human dopaminergic neuroblastoma cell line SH-SY5Y	Silybin A Silybin B Isosilybin A Silychristin A Silydianin 2,3-Dehydrosilybin A **2,3-Dehydrosilybin B**	[133]
Antiaggregation activity of Aβ in *C. elegans* CL4176 strain overproducing Aβ; inhibition of Aβ40 fiber formation by ThT assay	Aβ_40_ fiber formation kinetics measured by ThT assay; transgenic *Caenorhabditis elegans* CL4176 strain expressing human Aβ42 in the body-wall muscle	Silybin A **Silybin B** 2,3-Dehydrosilybin A 2,3-Dehydrosilybin B	[101]
Inhibition of fibrillation of Aβ_42_ monomers	Human dopaminergic neuroblastoma cell line SH-SY5Y challenged Aβ_42_; Aβ_40_ fiber formation kinetics measured by ThT assay	Silybin A Silybin B Silybin A-23-phosphotrehaloside Silybin B phosphotrehaloside	[102]
Inhibition of HEWL by fibril-induced cytotoxicity in SH-SY5Y cells	HEWL guanidine-induced fibrillation process assay (ThT); neuroblastoma cell line SH-SY5Y treated with HEWL fibrils	Silybin A **Silybin B**	[134]
Attenuating of nitrosative stress in both PC12 and HepG2 cells; increase in mitochondrial membrane potential and ATP levels	PC12 and human hepatoblastoma HepG2 cell lines	Silybin A	[135]
Inhibition of BRAF V600E kinase activity	HEK293T cells transfected with human Myc-DDK-tagged SMO; BRAF V600E mutant kinase assay	Silybin A Silybin B 2,3-Dehydrosilybin A **2,3-Dehydrosilybin B**	[136]
Inhibition of estradiol-17β-glucuronide and rosuvastatin uptake by the cells	Human embryonic kidney cell lines expressing drug transporters—HEK293-Mock, HEK293-OATP1B3, and HEK293-OATP1B1	Silybin A Silybin B Isosilibinin A Isosilybin B Silychristin Silydianin	[137]
Inhibition of Na^+^/K^+^-ATPase	Ouabain-sensitive Na^+^/K^+^-ATPase from porcine kidney outer medulla	Silybin AB Silychristin A **2,3-Dehydrosilychristin A** Silydianin **2,3-Dehydrosilydianin**	[138]
Inhibition of 3,3′,5-triiodothyronine (T3) transport by MCT8	Thyroid hormone transmembrane transporters in MCT8 -overexpressing MDCK1-cells; primary murine astrocytes expressing endogenous Mct8 but not MCT10-overexpressing MDCK1-cells	**Silychristin** Silydianin Isosilibinin A Isosilybin B Silybin AB	[139]
Dose-dependent inhibition of P-gp pump; sensitization of doxorubicin-resistant ovarian carcinoma; downregulating the MDR gene expression; inhibition of acetylcholinesterase	P-gp-Glo assay system; human ovarian carcinoma cell line resistant to doxorubicin (HOC/ADR, A2780/ADR); macrophages (RAW 264.7); acetylcholinesterase activity	Silybin A **Silybin B** 2,3-Dehydrosilybin A 2,3-Dehydrosilybin B Quercetin	[140]
Inhibition of P-glycoprotein and/or its expression; Sensitization of doxorubicin-resistant ovarian carcinoma; anti-inflammatory activity; Inhibition of ABC transporter expression	P-gp-Glo assay system; human ovarian carcinoma cell line resistant to doxorubicin (HOC/ADR, A2780/ADR); macrophages (RAW 264.7); ORAC assay	Silybin A **Silychristin A** Isosilychristin **2,3-Dehydrosilychristin A** Anhydrosilychristin	[141]
Tyrosinase inhibition	Mushroom tyrosinase with l-tyrosine or l-DOPA as substrates	**Silybin AB** Isosilybin A Isosilybin B Silychristin A Silychristin B Silydianin 2,3-Dihydrosilychristin	[142]
Inhibition of (*S*)-warfarin 7-hydroxylation with CytP450 isoenzyme CYP2C9	Human liver microsomes; recombinant CYP2C9	Silybin A **Silybin B** Isosilybin A Isosilybin B	[143]
Inhibition of midazolam 1’-hydroxylation with CYP3A	Human liver microsomes; human intestinal microsomes; recombinant human rCYP3A4 with midazolam as a substrate.	Silybin A Silybin B Isosilybin A Isosilybin B Silychristin Isosilychristin Silydianin Taxifolin	[144]
Inhibition of intestinal glucuronidation	Human liver microsomes; Human intestinal microsomes; HEK293-cells overexpressing UGT1A, UGT1A8, and UGT1A10 with 4-methylumbelliferone as a substrate	**Silybin A** Silybin B Isosilybin A Isosilybin B Silychristin Isosilychristin Silydianin	[145]
Increase in intracellular bilirubin concentration in HepG2 cells; downregulation of UGT1A1 mRNA expression in mice (liver), the elevation of serum bilirubin concentrations, decrease in lipoperoxidation in liver	Human hepatoblastoma HepG2 cells; C57BL/6 mice intraperitoneal or oral administration of tested compounds	Silybin A Silybin B Isosilybin A Isosilybin B Silychristin A Silydianin **2,3-Dehydrosilybin A** **2,3-Dehydrosilybin B** 2,3-Dehydrosilychristin 2,3-Dehydrosilydianin	[146]

^a^ Bold entries: the most active substances in the respective panel.

## Abbreviations

Aβ	Amyloid β
ABCA1	Transmembrane ATP-binding cassette transporter A1;
ADME	Absorption, distribution, metabolism, and excretion
AhR	Aryl hydrocarbon receptor
APX1	Ascorbate peroxidase
AR	Androgen receptor
AUC	Area under the curve
BRAF	Serine/threonine-protein kinase B-raf
Cdk2; Cdk4	Cyclin-dependent kinases
CYP	Cytochrome P-450
*d.e.*	Diastereomeric excess
DM1	Diabetes mellitus type 1
DM2	Diabetes mellitus type 2
DMF	Dimethylformamide
ECD	Electron circular dichroism
EGCG	*epi*-Gallocatechin-3-gallate
EtOAc	Ethyl acetate
ER	Estrogen receptors
FGT-1	Facilitative glucose transporter isoform 1
GLP-1	Glucagon-like peptide-1 production
GSH	Glutathione (reduced)
HDL	High-density lipoprotein
HEK293	Human embryonic kidney
HEWL	Hen egg-white lysozyme
HH	Human hepatoma
HPC	Human prostate carcinoma
HUVEC	Human umbilical vein endothelial cells
IR	Infrared spectroscopy
IT-TOF	Ion trap time-of-flight
IT	Ion trap
MCT8	Monocarboxylate transporter 8
MDCK1	Madin–Darby canine kidney
MDR	Multi-drug resistance
MS	Mass spectrometry
NMR	Nuclear magnetic resonance
Nrf2-HO-1/SOD2	Estrogen receptor-α-dependent Nrf2-heme oxygenase-1/superoxide Dismutase 2 antioxidative
PA	Prostate adenocarcinoma
PC12	Pheochromocytoma of the rat adrenal medulla
PPARγ	Peroxisome proliferator-activated receptor γ
Q-TOF	Quadrupole time-of-flight
SQ	Sector quadrupole
SULT	Sulfotransferases
TBME	*tert*-Butyl methyl ether
ThT	Thioflavin T
TLC	Thin-layer chromatography
UDP	Uridine diphosphate
UGT	UDP-glucuronosyl transferase.
UHPLC	Ultra high performance liquid chromatography
VLDL	Very low-density lipoproteins
